# *Candida albicans* Extracellular Vesicles Upregulate Nrg1 Transcription Repressor to Inhibit Self-Hyphal Development and Candidemia

**DOI:** 10.3390/ijms27010495

**Published:** 2026-01-03

**Authors:** Yu Wei, Yujie Zhou, Bolei Li, Zheng Wang, Binyou Liao, Jiannan Wang, Jingzhi Zhou, Yawen Zong, Ding Chen, Jiawei Shen, Yangyang Shi, Xuedong Zhou, Ga Liao, Lichen Gou, Zhuoli Zhu, Lei Cheng, Biao Ren

**Affiliations:** 1 State Key Laboratory of Oral Diseases, National Clinical Research Center for Oral Diseases, West China School of Stomatology, Sichuan University, Chengdu 610041, China; weiyu1101@foxmail.com (Y.W.); libolei7@scu.edu.cn (B.L.); wangzhengdentist@163.com (Z.W.); liaobinyou@126.com (B.L.); jiannan_wang@foxmail.com (J.W.); zhoujingzhi@stu.scu.edu.cn (J.Z.); zongywkq@foxmail.com (Y.Z.); chending1144@163.com (D.C.); sjw15531847709@163.com (J.S.); shiyangyang@stu.scu.edu.cn (Y.S.); zhouxd@scu.edu.cn (X.Z.); liaoga@hotmail.com (G.L.); goulichen1997@163.com (L.G.); zzl7507@126.com (Z.Z.); 2 Guangdong Provincial Key Laboratory of Stomatology, Guanghua School of Stomatology, Sun Yat-Sen University, Guangzhou 510055, China; zhouyujie0205@foxmail.com; 3 Tianfu Jiangxi Laboratory, Chengdu 641419, China

**Keywords:** *Candida albicans*, extracellular vesicles, hyphae, virulence, *NRG1*

## Abstract

*Candida albicans* is the most prevalent opportunistic pathogenic fungus in humans, and its extracellular vesicles (EVs) play crucial roles in its growth and pathogenesis. Previously, we found that *C. albicans* EVs at low levels could promote its growth. However, the effects of EVs when accumulated at high concentrations in *C. albicans* remain unclear. This study revealed that a high concentration of EVs inhibited hyphal development in *C. albicans* in a time-dependent manner. Transcriptome and RT-qPCR analyses showed that EVs significantly upregulated the transcription repressor *NRG1* and downregulated hyphal-specific genes in a laboratory strain and five clinical isolates, while EVs failed to repress *nrg1Δ/Δ* hyphae. Further experiments confirmed that EVs upregulated the upstream transcription factor *SKO1* (but downregulated *BRG1*) to increase *NRG1* expression, thereby inhibiting hyphal formation. Cargo proteins in EVs were key components that inhibited *C. albicans* hyphal growth. Additionally, EV-treated *C. albicans* showed improved mouse survival and reduced organ fungal burden in candidemia, but EVs did not attenuate virulence in *nrg1Δ/Δ*-infected mice. These results reveal that *C. albicans* EVs at high levels play an important role in its pathogenicity and highlight the potential for novel therapeutic strategies.

## 1. Introduction

Candidiasis and candidemia, predominantly caused by *Candida albicans* [1], represent major fungal infections. *C. albicans* ranks as the most prevalent opportunistic fungal pathogen [2,3,4], being designated a priority pathogen in the WHO’s first Fungal Priority Pathogens List (FPPL) [5]. It colonizes mucosal surfaces (oral, vaginal, gastrointestinal) as a commensal but transitions from a yeast to invasive hyphae in immunocompromised hosts (e.g., those with AIDS or undergoing chemotherapy), causing superficial or systemic infections [6,7].

This morphological plasticity, as a hallmark of *C. albicans* virulence, is regulated by a network of signaling pathways and transcription factors [7]. The regulation of hyphal development in *C. albicans* involves several pathways, such as the Cek-MAPK, cAMP-PKA, and Hog-MAPK pathways [8]. Key repressors of hyphal morphogenesis include the transcription factors Nrg1 and Sko1 [9,10]. *NRG1* binds to hyphal-specific gene promoters to suppress their expression during yeast-phase growth, but rapidly dissociates under hyphal-inducing conditions [11,12]. The overexpression of *NRG1* blocks germ tube formation, even under strong induction [13,14,15]. *SKO1* is also a yeast-to-hyphal transition-repressive transcription factor of *C. albicans*, and Sko1 may be regulated via the cAMP-PKA signaling pathway [16]. Sko1 was also reported to mediate the recruitment of the Tup1-Ssn6/Cyc8 complex to the promoter regions of certain Hog1-dependent genes in response to oxidative stress [17]. Active Hog1 represses the expression of *BRG1* via the transcriptional repressor Sko1; conversely, reduced *BRG1* expression promotes the expression of *NRG1*, a key repressor of *C. albicans* hyphal growth [9,10]. These regulatory cascades indicate the positive regulatory ability of *SKO1* on *NRG1* during hyphal development in *C. albicans*.

EVs are lipid-bilayer-enclosed nanoparticles (50–500 nm in diameter) secreted by all domains of life and carry proteins, lipids, nucleic acids, and metabolites [18,19]. In fungi, EVs are generated through endosomal sorting complex (ESCRT)-dependent and -independent pathways, with their cargoes varying under different environmental conditions [20,21]. Fungal EVs are typically isolated via differential ultracentrifugation, followed by characterization using nanoparticle tracking analysis (NTA), transmission electron microscopy (TEM), and proteomic profiling [22,23,24].

Fungal EVs have been shown to play important roles in pathogenesis [25,26]. Fungal-secreted EVs have been shown to be capable of modulating host innate immune responses by either activating the innate immune system to eliminate fungal infections or inhibiting macrophage phagocytosis and intracellular yeast killing by innate immune cells, thereby promoting the survival of fungal pathogens and persistent infections [27,28,29,30,31,32]. Some fungal EVs have been explored as vaccine formulations against fungal keratitis and systemic candidiasis, possibly due to the immunogenic components and proteins present in these EVs [22,33,34,35]. Beyond their protective actions against fungal infections, they have also been reported to promote fungal infections [36,37]. *Cryptococcus neoformans* EVs facilitate fungal cell crossing of the blood–brain barrier (BBB), thereby promoting brain infection [38]. EVs derived from *Sporothrix brasiliensis* induced an increase in the phagocytic index and fungal load in dendritic cells [39]. Current studies on fungal EVs have proven their important role in fungal infections and their interactions with host cells. However, these interactions, such as the balance between their dual roles in promoting fungal infection and activating the immune system, are complex, and many details remain unclear.

*C. albicans* EVs have critical impacts on growth and pathogenesis [24,40,41]. They could deliver the virulence factor candidalysin [42] and modulate fungal pathogenesis by regulating host immunity, activating macrophage cytokine production (IL-12, TGF-β, NO) and epithelial delivery of candidalysin [29,43], while potentially triggering cGAS-STING-mediated antifungal responses [44,45] and serving as vaccine candidates [34,46]. They also mediate interspecies interactions, such as enhancing stress tolerance in *Aspergillus* and *Paracoccidioides* [47], suppressing bacterial virulence (e.g., *E. faecalis*) [48], and facilitating inter-*Candida* biofilm formation through conserved proteins [49]. These EVs could also enhance the integrity of biofilm and the ability of drug isolation by delivering extracellular matrix components (such as dextran and manannan), and mediate the drug resistance of strains [21,50]. Recent studies have shown that EVs can inhibit biofilm formation via germ tube suppression (mediated through sesquiterpenes/fatty acids) [51]. We previously demonstrated that low concentrations of *C. albicans* EVs (15 μg/mL) strongly promoted the proliferation of yeast-form cells by activating the L-arginine/nitric oxide (NO) signaling pathway, which reduced intracellular reactive oxygen species (ROS) accumulation [52]. Despite these roles, the effects of *C. albicans* EVs accumulated at high levels on its pathogenesis remain unclear. Here, we identify a novel mechanism whereby EVs attenuate hyphal development by activating Nrg1 and reducing its pathogenicity in murine systemic candidiasis, advancing EV-based therapeutic strategies.

## 2. Results

### 2.1. Extracellular Vesicles Inhibited C. albicans Hyphal Development

**Figure 1:**
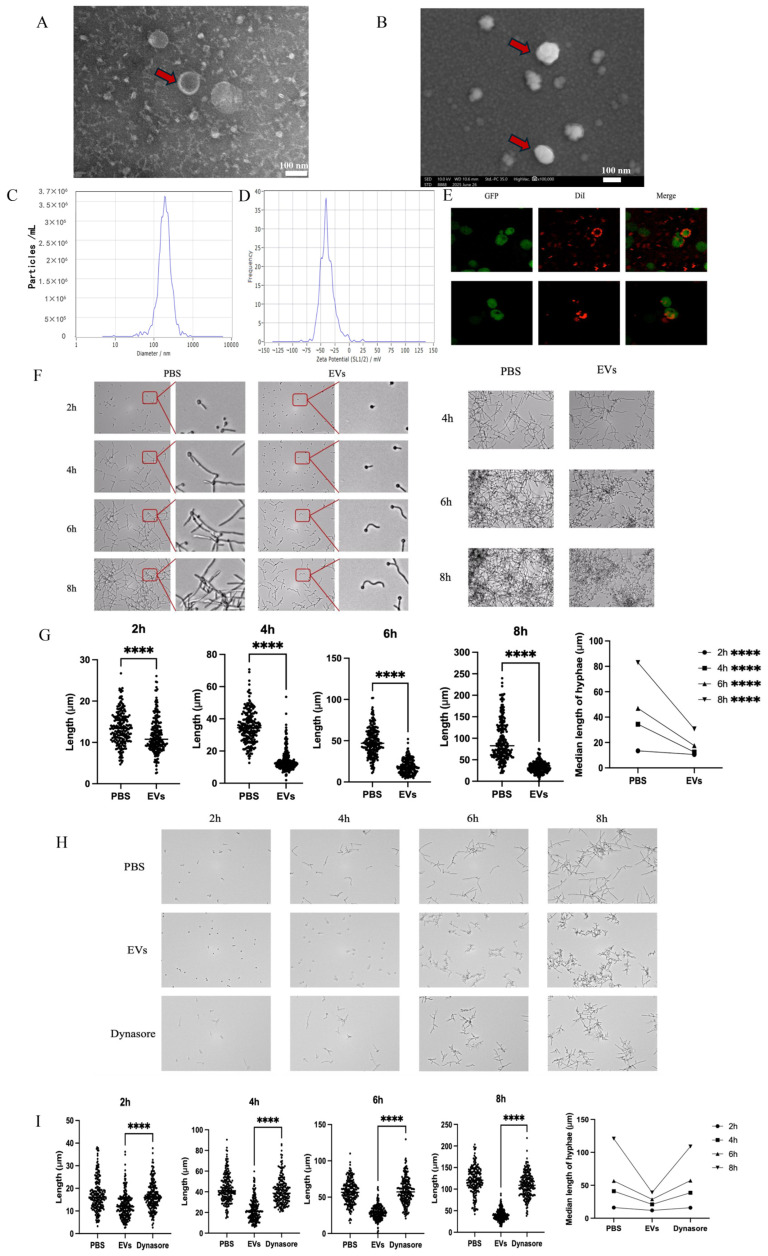
EVs inhibited the hyphal development of *C. albicans*. (**A**) TEM of *C. albicans* EVs. Scale bar, 1 μm. Red arrows indicate EVs with bilayer lipid membrane structure. (**B**) SEM of *C. albicans* EVs. Scale bar, 100 nm. Red arrows indicate EVs with bilayer lipid membrane structure. (**C**) Range of size distribution of *C. albicans* EVs measured by nanoparticle tracking analysis (NTA). (**D**) Zeta potential analysis of *C. albicans* EVs. (**E**) Confocal imaging of GFP-*C. albicans* incubated with EVs (*C. albicans* is labeled green, and EVs are labeled red). (**F**) The inhibitory effect of 60 μg/mL EVs on *C. albicans* hyphae was observed under a Cytation^TM^ 5 Cell imaging multimodal detector (BioTek Instruments, Inc., Winooski, VT, USA) at different time points (two **left panels**: 20×; two **right panels**: 10×, 30 °C). PBS served as the control. (**G**) Hyphal length was quantified at each time point. (**H**) Effect of 60 μg/mL EVs on hyphal development after inhibition of *C. albicans* endocytosis using Dynasore (30 °C). (**I**) Hyphal length was quantified at each time point after inhibition of *C. albicans* endocytosis using Dynasore (**** *p* < 0.0001).

According to observations made via transmission electron microscopy (TEM) and scanning electron microscopy (SEM), *C. albicans* EVs were confirmed as spherical nanoscale particles with a bilayer lipid membrane (Figure 1A,B). Nanoparticle tracking analysis (NTA) indicated that the major population of EV diameters ranged from 180 to 500 nm, with a small subpopulation up to nearly 1 μm (Figure 1C). Zeta potential analysis showed that EV potential was concentrated between −80 and 25 mV, with a peak value of −35.84 mV (Figure 1D).

When treating *C. albicans* cells with EVs, these EVs can contact the cell surface and enter the cells (Figure 1E). Moreover, hyphal development of *C. albicans* cells was significantly inhibited at all tested time points (Figure 1F). Hyphal length and distribution showed that hyphae were markedly repressed at 2 h, and the inhibitory effects became more pronounced with increasing treatment time (Figure 1G). Importantly, after 8 h treatment, most *C. albicans* cells were in yeast form (Figure 1F), indicating the strong and persistent inhibition of EVs on *C. albicans* hyphal development. Pretreating *C. albicans* with Dynasore to inhibit EV endocytosis significantly reversed hyphal inhibition by EVs (Figure 1H,I), suggesting that EV entry into fungal cells is critical for hyphal repression.

### 2.2. Extracellular Vesicles Shifted the Expression of Hyphal-Related Genes from C. albicans Transcriptome

**Figure 2:**
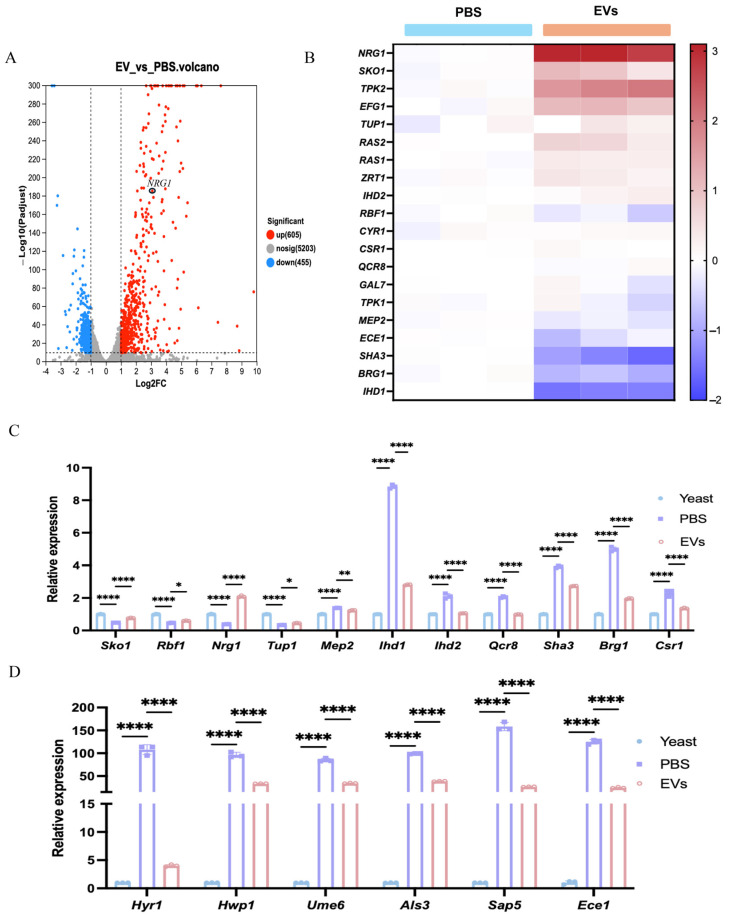
EVs regulated the expression of hyphal- and virulence-related genes in *C. albicans*. (**A**) Volcano plots of differentially expressed genes between 60 μg/mL EVs and PBS treatments (30 °C). (**B**) Heatmap of *C. albicans* hyphal-related gene expression from transcriptomics. (**C**) Differential expression of *C. albicans* hyphal-related genes analyzed via RT-qPCR. (**D**) EVs decreased the expression of *C. albicans NRG1*-regulated virulence genes, analyzed via RT-qPCR (**** *p* < 0.0001, ** *p* < 0.01, * *p* < 0.05).

To reveal the mechanisms by which *C. albicans* EVs impact hyphal development, the transcriptome of EV-treated *C. albicans* was sequenced and analyzed. According to the transcriptome analysis, *C. albicans* EVs upregulated 605 genes and downregulated 455 genes (Figure 2A). Notably, a majority of hyphal-negative regulatory genes, including *NRG1* and *SKO1*, were significantly upregulated, while hyphal-positive regulatory genes, such as *BRG1* and *IHD1*, were significantly downregulated (Figure 2B). RT-qPCR analysis also indicated that, along with the hyphal formation, hyphal- and virulence-related genes were significantly upregulated (PBS group vs. yeast), while EV treatment significantly reduced the expression of these genes due to its inhibitory effects on hyphal development (Figure 2C,D). These results suggest that EVs enhanced the expression of hyphal-negative regulatory genes while suppressing positive regulatory genes, thereby inhibiting *C. albicans* hyphal development. *NRG1*, a transcription repressor of *C. albicans* hyphal formation, was significantly upregulated among these genes (Figure 2B,C), indicating that EVs might mainly upregulate *NRG1* to repress *C. albicans* hyphal development. Compared to the PBS group, the upregulation of *NRG1* via *C. albicans* EVs then caused significant downregulation of several *NRG1*-repressed virulence genes, including *ALS3*, *SAP5*, *HWP1*, *HYR1*, *ECE1*, and *UME6* (Figure 2D), suggesting that EVs inhibited *C. albicans* hyphal development and reduced virulence.

### 2.3. Extracellular Vesicles Inhibited C. albicans Hyphal Development Through the Upregulation of NRG1

**Figure 3:**
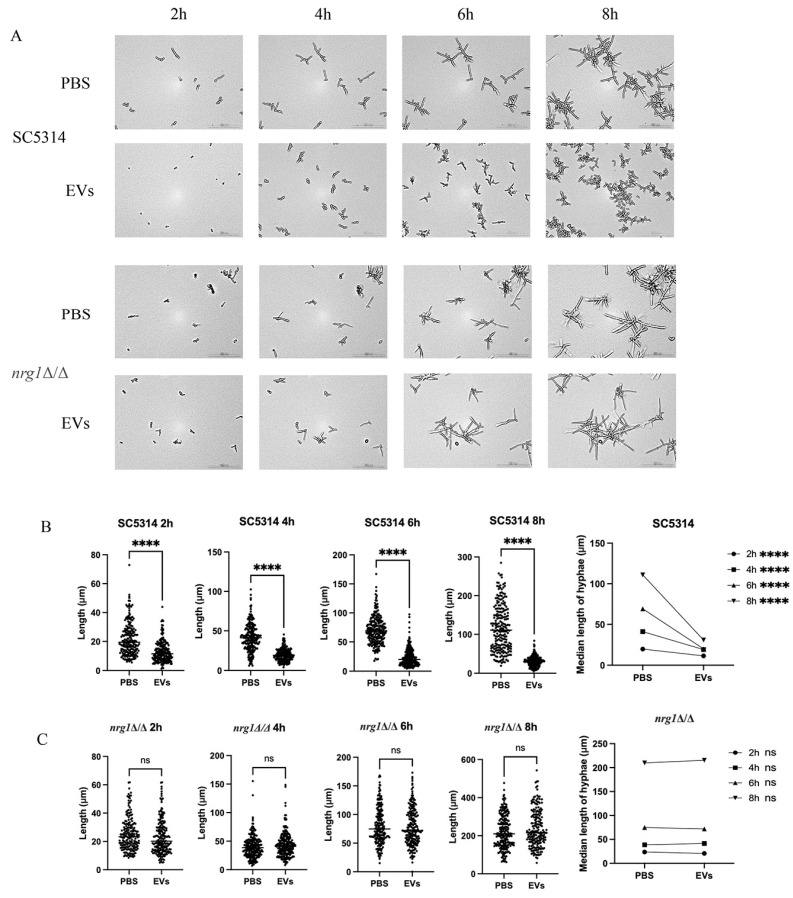
EVs upregulated *NRG1* to inhibit *C. albicans* hyphal development. (**A**) The inhibitory effects of 60 μg/mL EVs on wild-type strain (SC5314) and *nrg1Δ/Δ* hyphae were observed under a Cytation™ 5 Cell imaging multimodal detector at different time points (20×, 30 °C). (**B**,**C**) Hyphal length was quantified at each time point (**** *p* < 0.0001, ns: *p* > 0.05).

An *NRG1* null mutant was then employed to validate the effects of *C. albicans* EVs on hyphal development through this transcription repressor. EVs were able to repress the hyphae of SC5314 (*NRG1*/*NRG1*) but failed to inhibit mycelial growth of *nrg1Δ/Δ* at different time points (Figure 3A). EVs also showed no significant effects on the hyphal length of *nrg1Δ/Δ* at different time points (Figure 3B,C), indicating that EVs upregulated *NRG1* to repress *C. albicans* hyphal development.

### 2.4. Extracellular Vesicles Upregulated NRG1 to Inhibit the Hyphal Development of Clinical C. albicans Isolates

**Figure 4:**
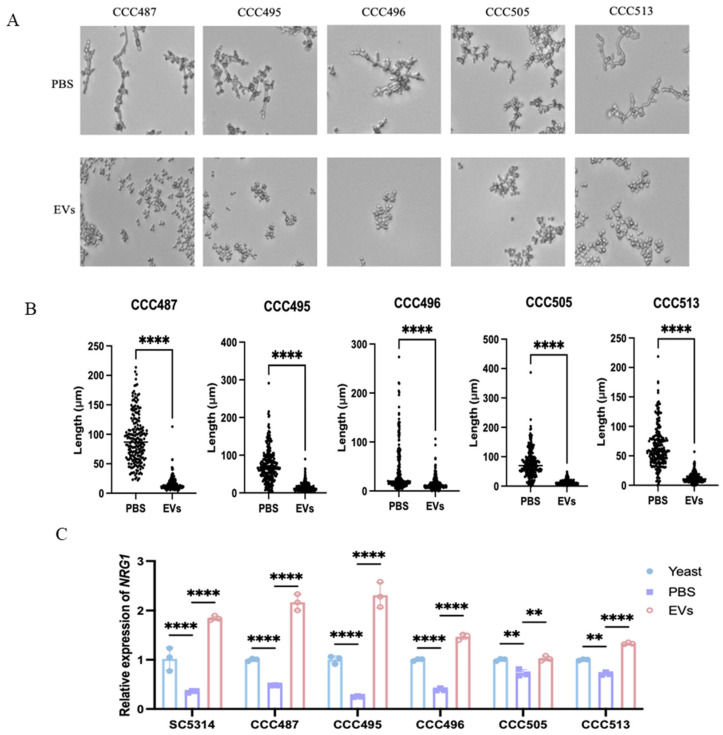
EVs inhibited the hyphal development of clinical *C. albicans* isolates. (**A**) The hyphal-inhibitory effects of 60 μg/mL *C. albicans* SC5314 EVs on clinical *C. albicans* isolates were observed under a Cytation™ 5 Cell imaging multimodal detector (20×, 30 °C). (**B**) Hyphal lengths were quantified from different clinical isolates treated with *C. albicans* SC5314 EVs or PBS. (**C**) Expression of *NRG1* in clinical *C. albicans* isolates after *C. albicans* SC5314 EV treatment via RT-qPCR (**** *p* < 0.0001, ** *p* < 0.01).

To further evaluate the hyphal-inhibitory effects of EVs, five clinical *C. albicans* isolates were then employed. *C. albicans* SC5314 EVs effectively inhibited hyphal development of all clinical isolates, including CCC487, CCC495, CCC496, CCC505, and CCC513 (Figure 4A), while their hyphal lengths were also significantly decreased by EVs (Figure 4B). RT-qPCR revealed that compared to the PBS group, EVs could also increase *NRG1* expression in these clinical isolates, similar to the laboratory strain SC5314 (Figure 4C). This suggests that EVs could repress the hyphal development of clinical *C. albicans* isolates by upregulating the transcription repressor *NRG1*.

### 2.5. Extracellular Vesicles Mediated Upregulation of SKO1 Correlates with Enhanced NRG1 Expression

**Figure 5:**
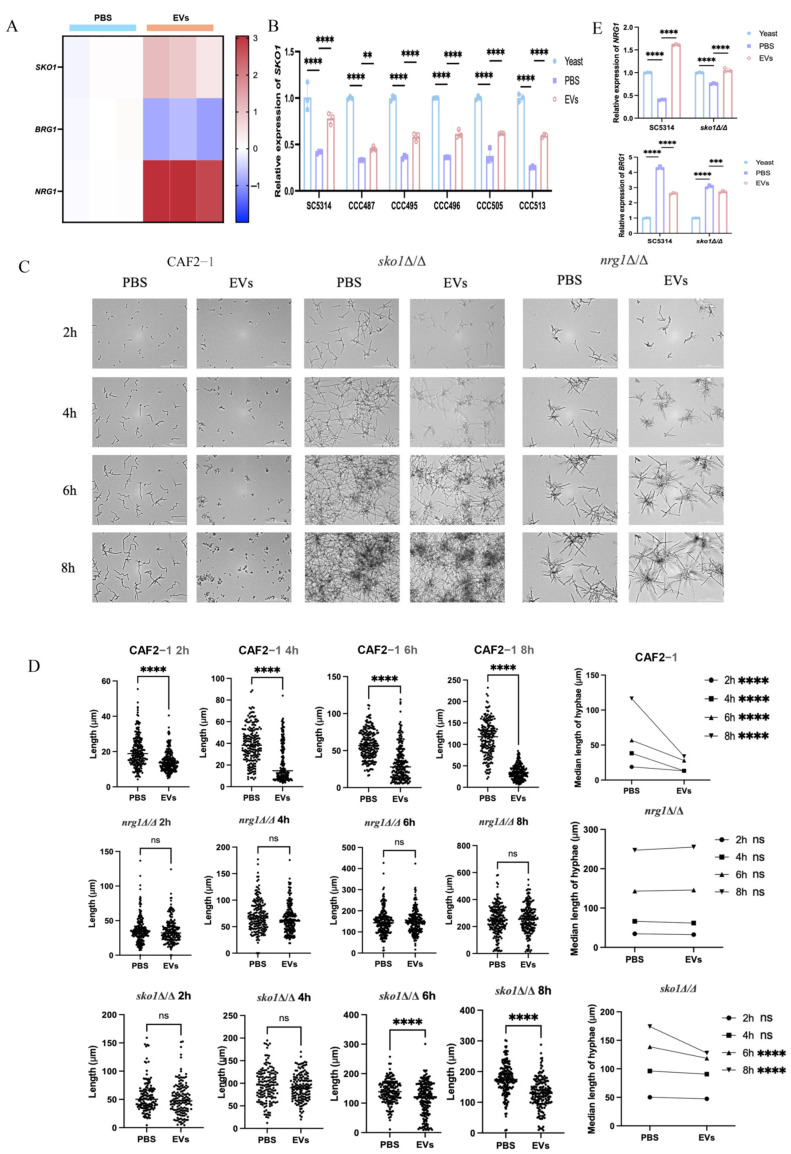
EVs upregulated *SKO1* to increase the expression of *NRG1* and repress *C. albicans* hyphal development. (**A**) Heatmap of *SKO1*, *BRG1*, and *NRG1* expression based on transcriptomics. (**B**) Relative expression of *SKO1* in *C. albicans* laboratory strain SC5314 and clinical isolates. (**C**) The inhibitory effects of 60 μg/mL EVs on the hyphae of wild-type CAF2-1, *sko1Δ/Δ*, and *nrg1Δ/Δ*, observed under a Cytation™ 5 Cell imaging multimodal detector at different time points (20×, 30 °C). (**D**) Hyphal lengths of wild-type CAF2-1, *sko1Δ/Δ*, and *nrg1Δ/Δ*, quantified at different time points. (**E**) Relative expression of *NRG1* and *BRG1* in wild-type SC5314 and *sko1Δ/Δ* via RT-qPCR (**** *p* < 0.0001, *** *p* < 0.001, ** *p* < 0.01, ns: *p* > 0.05).

To further investigate the mechanism by which EVs upregulate *NRG1* expression, we found that the expression of the transcription factor *SKO1* was also significantly upregulated, while the transcription factor *BRG1* was significantly downregulated, according to transcriptome analysis (Figure 5A). Since *NRG1* could be downregulated via *BRG1* and Sko1 could downregulate *BRG1*, as previously reported [9,53], we proposed that EVs possibly upregulated *SKO1* to increase *NRG1* expression. To confirm this hypothesis, *SKO1* expression from *C. albicans* SC5314 and clinical isolates was first measured. The results showed that compared to the PBS group, EVs significantly increased *SKO1* expression in these strains (Figure 5B). We then employed the *sko1Δ/Δ* mutant and observed that EVs significantly attenuated their hyphal-inhibitory effects—a phenotype similar to that seen in *nrg1Δ/Δ* mutants (Figure 5C). EVs also reduced inhibitory effects on *sko1Δ/Δ* hyphal lengths at different time points (Figure 5D). Moreover, EVs significantly increased the expression of *NRG1* in the wild-type strain SC5314 but significantly reduced *NRG1* upregulation in *sko1Δ/Δ*. EVs also significantly inhibited *BRG1* expression in the wild-type strain SC5314 but reduced *BRG1* downregulation in *sko1Δ/Δ* (Figure 5E). These results suggest that EVs could upregulate *SKO1* to enhance the expression of the *NRG1* repressor and subsequently inhibit *C. albicans* hyphal growth.

### 2.6. Protein Components Within EVs Contributed to Hyphal-Inhibitory Effects

**Figure 6:**
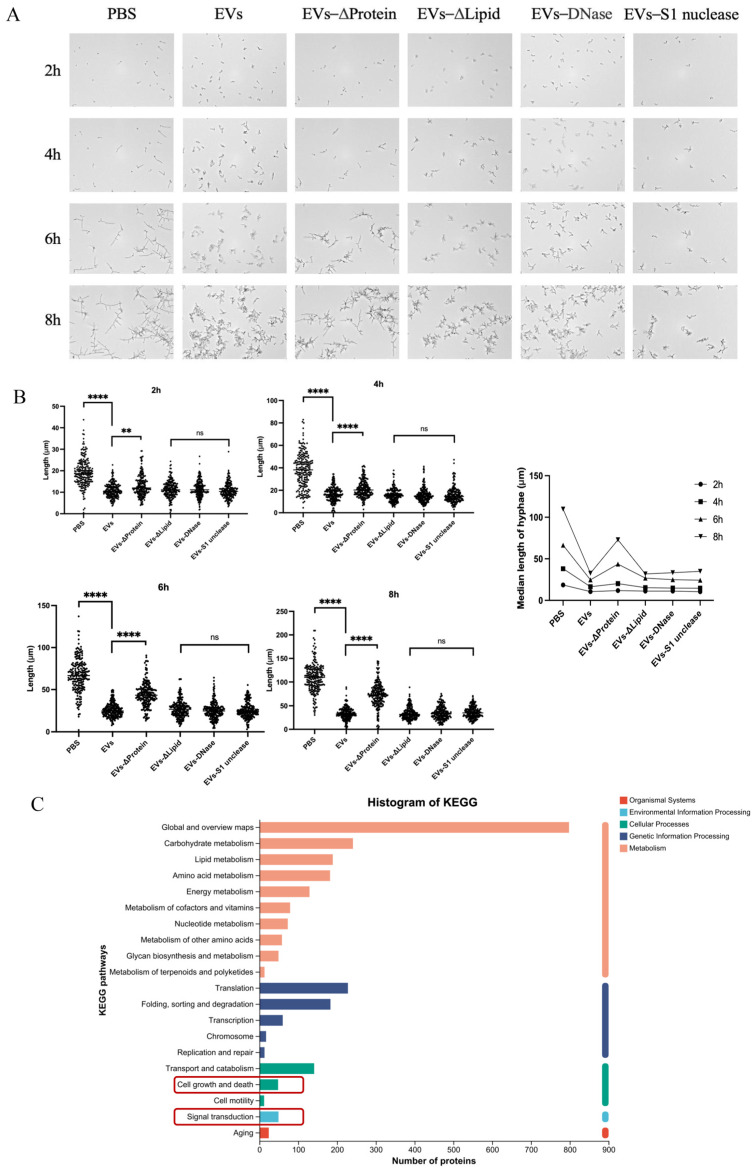
Protein components within EVs inhibit hyphal growth. (**A**) The inhibitory effects of 60 μg/mL EVs with depletion of different components on hyphae observed under a Cytation™ 5 Cell imaging multimodal detector at different time points (20×, 30 °C). (**B**) Hyphal lengths quantified at different time points (**** *p* < 0.0001, ** *p* < 0.01, ns: *p* > 0.05). (**C**) KEGG analysis of EV proteins identified via full-spectrum sequencing.

To identify the key components of EVs that mediated hyphal development suppression, we selectively depleted different EV constituents using the following enzymatic treatments: protease K (to degrade proteins), sodium deoxycholate (to disrupt lipids), dsDNase (to digest double-stranded DNA), and S1 nuclease (to remove single-stranded DNA and non-dsRNA). Protein depletion significantly reduced the inhibitory effect of *C. albicans* EVs on hyphal development (Figure 6A,B). By sequencing the global proteomic profiling of EVs and conducting KEGG pathway enrichment analysis (Figure 6C), notably, proteins associated with hyphal regulation, including Efg1, Ras1, Tup1, Cdc42, Cdc28, Gpa2, Yck2, Rho1, and Ndt80, were identified (Table S2) and enriched in the “cell growth and death” and “signal transduction” pathways (Figure 6C). Additionally, some other hyphal-related proteins, such as Hyr1, Ihd1, Mep2, and Gal7, were also identified in EVs (Table S2), while their corresponding expressions were significantly downregulated according to the *C. albicans* transcriptome (Figure 2B). Based on the observed downregulation of hyphal-regulatory transcripts and the proteomic identification of their fragmented counterparts in EVs, we infer that EVs may deliver fragmented peptides derived from these proteins to competitively inhibit the transcription of their endogenous counterparts, thereby disrupting hyphal morphogenesis in *C. albicans*.

### 2.7. Extracellular Vesicles Reduced the Pathogenesis of C. albicans Through NRG1 in Candidemia Mice

**Figure 7:**
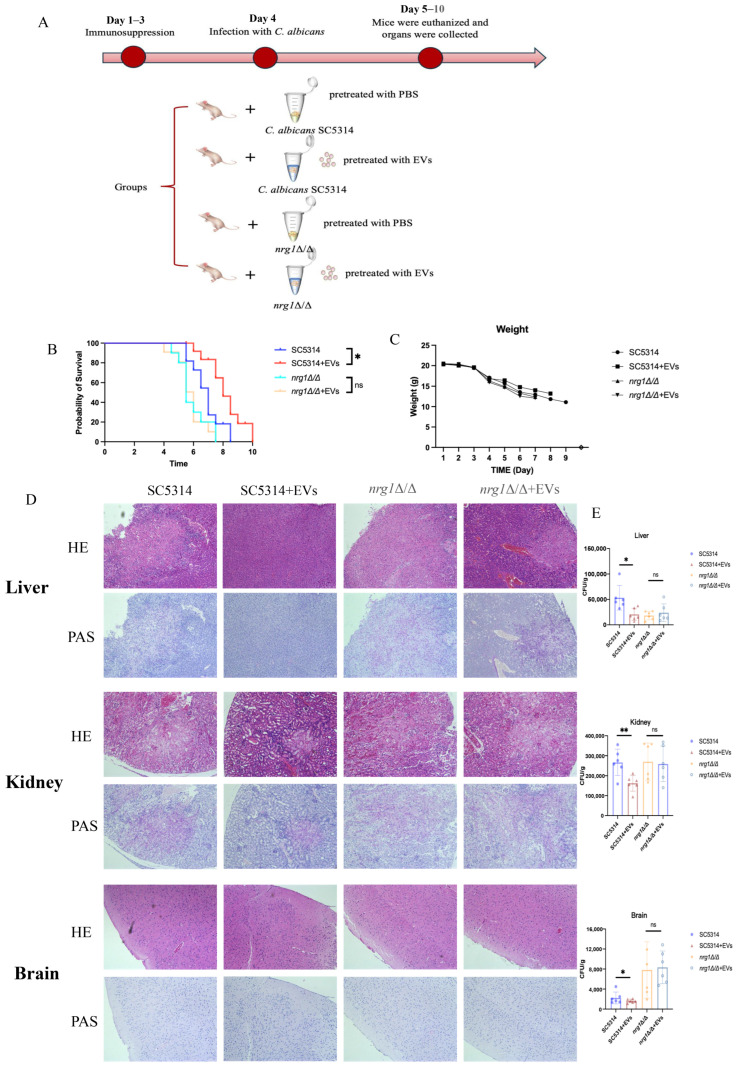
EVs reduced *C. albicans* pathogenesis in mice with candidemia. (**A**) Procedures for animal experiments on candidemia. (**B**) Survival curves of mice infected with *C. albicans* SC5314 and *nrg1Δ/Δ* pretreated with PBS or EVs. (**C**) Body weight changes in mice infected with *C. albicans* SC5314 and *nrg1Δ/Δ* pretreated with PBS or EVs. (**D**) Histopathologic assessment of liver, kidney, and brain tissues via H&E and PAS staining. (**E**) Fungal burden in liver, kidney, and brain tissues of *C. albicans*-infected mice (** *p* < 0.01, * *p* < 0.05, ns: *p* > 0.05).

Since EVs were able to inhibit *C. albicans* hyphae, a candidemia murine model was then established to further investigate the impact of EVs on *C. albicans* pathogenesis. Both SC5314 and *nrg1Δ/Δ* strains were pretreated with either PBS or EVs before being used to infect mice (Figure 7A). Mice infected with *nrg1Δ/Δ* mutants began to die on day 5, and none survived beyond day 8; EVs showed no effects on *nrg1Δ/Δ* mutant pathogenesis (Figure 7B). In contrast, mice infected with the SC5314 strain showed prolonged survival compared to those infected with *nrg1Δ/Δ* mutants; notably, mice infected with EV-pretreated SC5314 showed the longest survival (Figure 7B), although their weight did not show a significant difference during infection (Figure 7C). Afterward, liver, kidney, and brain tissues were collected for histological and CFU analysis. Histological analyses using HE and PAS illustrated significant reductions in inflammation and diminished fungal invasion in liver, kidney, and brain tissues of mice infected with EV-treated SC5314 (Figure 7D). Briefly, in the liver tissues of mice infected with EV-treated *C. albicans* SC5314, the contours of the liver lobules were largely preserved, with only small abscesses occasionally observed (Figure 7D). In contrast, the liver tissue structures of mice in the other groups exhibited disordered abscess structures and extensive infiltration of inflammatory cells (Figure 7D). PAS staining of the liver indicated that EV-treated SC5314 infection reduced fungal invasion (Figure 7D). Similarly, in the kidney tissues of mice infected with EV-treated SC5314, glomeruli and renal tubules remained clearly visible, while the kidney tissues of mice in the other groups exhibited abscesses, tissue rupture, and necrosis (Figure 7D). PAS staining of the kidneys also indicated that EV-treated SC5314 infection reduced the fungal invasion (Figure 7D). This is possibly due to the presence of the blood–brain barrier. In the brain tissue, there were no significant differences in inflammation; however, PAS staining indicated fungal invasion in the brain tissues of mice infected with SC5314 treated with or without EVs (Figure 7D). By directly counting *C. albicans* CFUs in tissues, EV treatment significantly reduced fungal burdens in the liver, kidneys, and brain (Figure 7E). Compared with SC5314-infected mice, EV treatment showed no significant impact on mice infected with the *nrg1Δ/Δ* strain, including liver, kidney, and brain inflammation and fungal invasion (Figure 7D). Moreover, CFU counts also showed that EVs did not affect fungal burden in *nrg1Δ/Δ*-infected tissues (Figure 7E), indicating that EVs reduce *C. albicans* pathogenesis by inhibiting hyphal development through the upregulation of *NRG1* repressor.

## 3. Discussion

**Figure 8:**
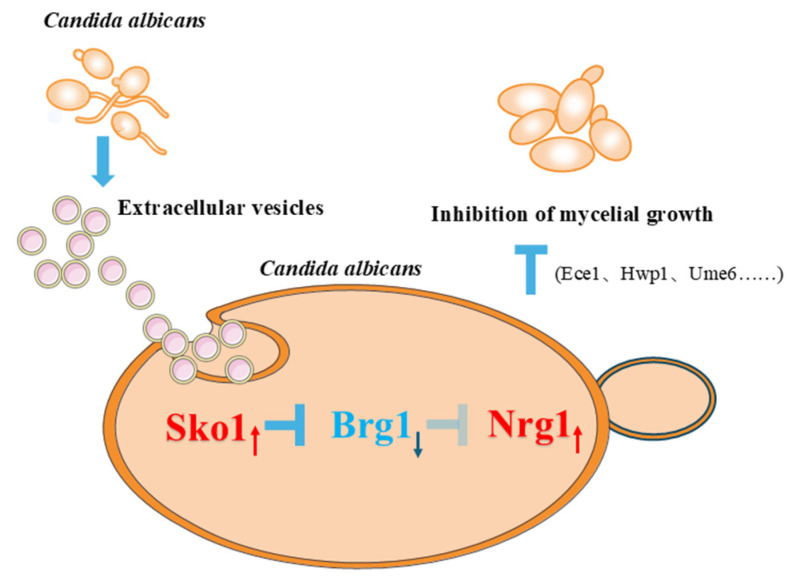
EV-mediated inhibition of hyphal development involves *SKO1*, *BRG1*, and *NRG1* regulators. Red arrows represent up-regulated genes and blue arrows represent down-regulated genes.

*C. albicans*, a predominant opportunistic pathogen, causes superficial infections (oral mucositis, vaginitis) and systemic candidemia [54]. Its extracellular vesicles (EVs) critically regulate interspecies communication and pathogenesis [24]. Building on our prior discovery that EVs promote fungal proliferation via L-arginine/NO-mediated reduction of ROS and apoptosis [52], this study elucidates a counter-regulatory mechanism: EVs suppress hyphal morphogenesis and systemic virulence by upregulating *SKO1* to enhance the repressor activity of Nrg1, ultimately attenuating candidemia (Figure 8). Combined with our previous study, EVs at different levels could show varied actions. EVs at low levels (15 μg/mL) mainly promote fungal cell proliferation by upregulating the L-arginine-NO pathway, while at high levels (60 μg/mL), *C. albicans* EVs primarily repress hyphal development by upregulating the *NRG1* transcriptional repressor. These mechanisms suggest the important role EVs play in *C. albicans* growth and pathogenesis. Currently, there are no reports about the accurate concentrations of *C. albicans* EVs in normal states or during systemic candidiasis due to the limitations of quantitation technology on *C. albicans* EVs in vivo, while the EV concentrations (15 and 60 μg/mL) used in our studies were rationally selected based on the reported range (5–80 μg/mL) in published fungal EV studies, and the fungal inoculum for EV extraction was consistent with that of mouse systemic candidiasis models, which alleviates the current limitation as much as possible.

Hyphal morphogenesis is essential for *C. albicans* virulence, enabling nutrient acquisition, tissue invasion, and expression of adhesins/hydrolases (Als3, Sap5, Hwp1) [55,56,57,58]. While polysaccharides inhibit hyphae via Nrg1 transcriptional control [59], we demonstrate that *C. albicans* EVs suppress hyphal development primarily through cargo-protein-mediated *NRG1* upregulation. Crucially, EVs concurrently downregulate virulence effectors—including candidalysin (*ECE1*-encoded) [42,60]—and enhance Nrg1 repressor activity in clinical isolates, attenuating systemic pathogenicity. Although EVs harbor polysaccharides [29,61], their role in hyphal inhibition remains uncharacterized; further investigation may elucidate interactions with the Nrg1 pathway, thereby informing the development of novel anti-virulence strategies.

*SKO1* is also a hyphal-negative regulatory gene. Additionally, active Hog1 represses *BRG1* gene expression via the transcriptional repressor Sko1; in turn, decreased *BRG1* expression can promote *NRG1* gene expression [8,9,10,53]. Our results indicated that *C. albicans* EVs upregulated *SKO1* and downregulated *BRG1*, thereby activating the *NRG1* repressor and inhibiting *C. albicans* hyphal development and pathogenesis (Figure 8). However, EVs failed to inhibit hyphal growth and pathogenesis in *sko1Δ/Δ* and *nrg1Δ/Δ*, indicating the critical role of this pathway in EV-mediated inhibition. These results also indicate that the *SKO1* and *NRG1* regulatory pathway is a promising target for developing anti-virulence agents against *C. albicans* infections. The inhibitory effect of EVs may involve multiple pathways because, unlike *nrg1Δ/Δ*, EVs also inhibited *sko1Δ/Δ* hyphal length at 6 and 8h. This suggests that *SKO1* knock-out partially restored the effects of EVs, and *NRG1* plays a stronger regulatory role. Other factors may regulate *BRG1* and *NRG1* beyond *SKO1*, such as Ngs1 [62] and cross-regulation between *BRG1* and *NRG1.* Previous studies have shown that active Hog1 represses *BRG1* expression through the transcriptional repressor Sko1 [9]; however, our transcriptomic data showed no significant change in *HOG1* expression (fold change = 0.97, *p* = 0.68) after treatment with EVs, suggesting that *HOG1* may not be a key factor in the impact of EVs on hyphal development. Other pathways, such as the cAMP-PKA [16] and Psk1-Rlm1 pathways [63,64], could activate *SKO1* expression independently of *HOG1*, potentially contributing to the actions of EVs.

In the current study, hyphal induction was performed at 30 °C, as our preliminary experiments revealed that the hyphal-inhibitory effect of EVs was stronger at this temperature than at 37 °C. This phenomenon might be partially due to temperature-dependent effects on *BRG1* expression. Lu et al. showed that induction at 37 °C activates *BRG1* expression, which may reduce *NRG1*-mediated downregulation of *BRG1* [53]. In contrast, at 30 °C, *BRG1* is expressed at low levels, which may promote the downregulation of *BRG1* through EV-activated *NRG1*.

EVs contain various components, and our findings highlight that the protein components within *C. albicans* EVs contribute to their hyphal repression activity. The major bioactive components responsible for hyphal inhibition may vary due to the different host strains and their respective culture media. For example, Honorato et al. found that nonpolar lipids from *C. albicans* 90,028 EVs cultured in a Sabouraud medium (a complex medium containing peptone and 4% glucose) repressed the yeast-to-hypha transition [51], while in our study, proteins from the *C. albicans* SC5314 EVs cultured in a YNB medium (a chemically defined medium with 2% glucose) showed strong inhibition of hyphal development. Despite this, both studies collectively demonstrated that *C. albicans* EVs inhibit its own hyphal growth, highlighting the important roles of EVs in *C. albicans* growth and pathogenesis. Our proteomic analysis revealed an enrichment of hyphal regulatory proteins, including Efg1, Ras1, and Tup1, which are central proteins from the cAMP-PKA and MAPK signaling pathways [65]. Nrg1 is known to inhibit hyphal genes by recruiting the Tup1-Cyc8 complex [66,67]. Sko1 modulates cellular responses to osmotic stress, which can intersect with morphogenetic signaling [68]. Based on the proteomic identification of Tup1 fragments within *C. albicans* EVs and the concomitant downregulation of hyphal-related transcripts (*EFG1*, *RAS1*), EV-delivered Tup1 peptides might competitively disrupt Nrg1-Tup1 complex formation. This interference would destabilize transcriptional repression circuits, thereby triggering suppression of hyphal-specific genes. Concomitantly, the observed downregulation of *SKO1*-associated transcripts (GPA2, *YCK2*) in the fungal transcriptome and the proteomic detection of Gpa2/Yck2 within EVs suggest that EV cargo containing these proteins might interfere with stress-responsive pathways converging on Sko1-mediated morphogenesis. These findings indicate that EVs may act as decoys that deliver various proteins or peptides from critical hyphal regulators, thereby affecting the hyphal differentiation network. Future studies should validate the direct interactions between EV-derived peptides and the hyphal differentiation network to elucidate their role in fungal pathogenesis.

It is well known that hyphae are important for host immune cells to recognize *C. albicans* and activate host antifungal immunity [69]. *C. albicans* secretes a large number of EVs during the infection process, which can regulate yeast proliferation [52] and hyphal development, potentially promoting yeast cell colonization and the escape of *C. albicans* from immune cells. This may be a protective, fitness-enhancing strategy that promotes *C. albicans* survival in hosts. However, the effects of *C. albicans* EVs on fitness require further evaluation. The ability of *C. albicans* EVs to reduce *C. albicans* virulence and pathogenesis—especially in clinical isolates—combined with their previously reported potential to act as vaccines against fungal infections [34,46] indicate their dual roles and practical applications in combating fungal infections.

## 4. Materials and Methods

### 4.1. Strains and Cultural Conditions

*Candida albicans* SC5314 (ATCC MYA-2876), wild-type strain (CAF2-1, WT) with *ura3* knocked out from *C. albicans* SC5314 [70], *sko1Δ/Δ* [71], and GFP-labeled *C. albicans* [72] were stored in the State Key Laboratory of Oral Diseases. *C. albicans* clinical strains were isolated from patients in the Department of Respiratory Medicine, West China School, Sichuan University.

CRISPR/Cas9 and the plasmid pV1524 [73] (Addgene, Watertown, MA, USA) were employed to construct the *C. albicans NRG1* gene deletion strain (*nrg1Δ/Δ*). Briefly, 20 bp sgRNA primers (sgRNA/F-ACTTTAGAAGCTTCACATGT and sgRNA/R-GTAAGAAGTTCGTTAAACGA) were ligated into the Esp3I restriction site of the pV1524 vector. The respective 500 bp upstream and downstream homology arms of the *NRG1* gene, which served as repair templates for CRISPR/Cas9-mediated gene editing, were introduced into the SacII restriction site of pV1524. A PacI restriction site was included in the repair template to facilitate linearization. The resulting plasmid was then transformed into *C. albicans* SC5314 via the LiAc/SS carrier DNA/PEG method [74,75]. *C. albicans* SC5314 cells and linearized plasmid (via PacI enzyme) were added to the prepared transformation Master Mix and incubated at 30 °C for 1 h, followed by a 15 min heat shock at 42 °C. The transformed cells were placed in YPD media containing 250 µg/mL nourseothricin (Werner BioAgents, Jena, Germany) and grown at 30 °C for 48 h to obtain the *nrg1Δ/Δ* strain. Subsequently, the *nrg1Δ/Δ* strain was verified via PCR (Figure S1).

All strains were grown on YPD plates (4 g yeast extract, 8 g anhydrous glucose, 8 g peptone, 8 g agar dissolved in 400 mL deionized water) at 35 °C overnight. For treatment with EVs, colonies of all strains were taken separately, placed in PBS, adjusted to a final concentration of 1 × 10^6^ CFU/mL, placed in RPMI 1640 (Gibco, Shanghai, China), and incubated at 30 °C for 24 h.

### 4.2. Isolation of Extracellular Vesicles

EVs from *C. albicans* were isolated using the ultracentrifugation method as reported before [21,35,52]. Briefly, *C. albicans* was inoculated on YPD plates for 24 h and incubated overnight at 30 °C. Then, fungal cells were seeded in 1 L of YNB (Solarbio, Beijing, China) medium at a final concentration of 1 × 10^6^ CFU/mL in conical flasks and incubated in a shaking incubator at 30 °C with agitation at 150 rpm for 72 h. Following cultivation, the supernatant was collected via centrifugation at 15,000× *g* for 30 min at 4 °C using a high-speed centrifuge (JA-10 rotor, Beckman, Fullerton, CA, USA). The supernatant was filtered through a 0.45 μm filter (Millipore, MA, USA) and concentrated to 100 mL through a VIVAFLOW 200 ultrafiltration membrane (Sartorius, Goettingen, Germany). The concentrated solution underwent additional centrifugation for 120 min at 100,000× *g* and 4 °C (JA-24.38 rotor, Beckman, Fullerton, CA, USA), yielding precipitates identified as EVs. Finally, after the EV precipitate was resuspended in PBS, it was filtered through a 0.22 µm filter (Millipore, Billerica, MA, USA) to ensure that all fungal cells were removed.

### 4.3. Characterization of EVs

Scanning electron microscopy: EVs were immobilized on glass slides (4 °C, overnight), gradient-dehydrated in an ethanol series, sputter-coated with a Au/Pd alloy, and subsequently visualized via microscopy (FEI Company, Hillsboro, OR, USA) [52]. Transmission electron microscopy: EVs were adsorbed on copper grids (10 min), negatively stained with 1% phosphotungstic acid (2 min), and imaged (FEI Company, Hillsboro, OR, USA) [76].

Particle size distribution of EVs: EVs were diluted 1:1000 in PBS (pH 7.4) to obtain 20–100 particles/frame and analyzed using a Malvern NanoSight (Malvern Panalytical, Malvern, Worcestershire, UK) (camera level 16, threshold 5; 25 ± 0.5 °C) with 5 × 60 s videos. Data were processed via NTA v3.4 and calibrated with 100 nm standards [51].

Zeta potential: EVs were diluted to 0.1 mg/mL in 1 mM NaCl (pH 7.0) and measured on ZetaView PMX120 (Particle Metrix GmbH, Memmingen, Germany) (488 nm laser; 25 ± 0.1 °C) using the Smoluchowski model. Three replicates (fifteen cycles each) were performed in folded capillary cells and calibrated with a −50 mV standard.

### 4.4. Confocal Imaging

EVs were labeled with 20 μM DiI (APExBIO, Houston, TX, USA) in PBS (RT, 2 h; 37 °C, 30 min, dark), then ultracentrifuged (100,000× *g*, 2 h) to remove excess dye. GFP-expressing *C. albicans* (1 × 10^5^ CFU/mL) was co-cultured with DiI-EVs (60 μg/mL) in RPMI 1640 (30 °C, 6 h). Cells were fixed with 4% PFA and imaged via confocal microscopy (Leica TCS SP8, Leica Microsystems GmbH, Wetzlar, Germany, 60× oil objective) with dual-channel acquisition: GFP 488 nm/500–550 nm, DiI 561 nm/570–620 nm, Z-stacks acquired at 0.5 μm intervals.

### 4.5. Real-Time Imaging and Distribution of C. albicans Hyphal Length

*C. albicans* at 1 × 10^5^ CFU/mL in RPMI 1640 (Gibco, Shanghai, China) were treated with 60 μg/mL EVs at the same time in a 96-well plate and incubated on a Cytation™ 5 Cell imaging multimodal detector (Biotek, VT, USA). The temperature was set to 30 °C, and continuous operation was maintained for 24 h, capturing images every 15 min. Hyphal length and distribution were determined using ImageJ software (Version 1.8.0.172, National Institutes of Health, Bethesda, MD, USA) for pixel quantification. More than 200 single cells were counted for each sample.

### 4.6. Dynasore-Treated C. albicans

*C. albicans* (1 × 10^5^ CFU/mL) were pre-incubated with 80 μM Dynasore (APExBIO, Houston, TX, USA, dissolved in 0.1% DMSO) in RPMI 1640 medium for 30 min (37 °C). This was followed by centrifugation to remove the effects of Dynasore in the supernatant. A vehicle control (0.1% DMSO alone) and an untreated group (no Dynasore/DMSO) were included. Then, *C. albicans* were treated with EVs for the real-time imaging shots described above.

### 4.7. Transcriptome Analysis

Transcriptome sequencing was performed as previously reported [52,77]. Total RNA was extracted from *C. albicans* cells (1 × 10^6^ CFU/mL) treated with 60 μg/mL EVs or the PBS control (RPMI 1640, 30 °C, 6 h) using TRIzol Reagent (Invitrogen, Carlsbad, CA, USA), followed by DNase I (Takara, Tokyo, Japan) digestion. RNA quality was verified (Agilent 2100 Bioanalyzer; NanoDrop ND-2000) with the following criteria: OD_260/280_ = 1.8–2.2, OD_260/230_ ≥ 2.0, RIN ≥ 6.5, 28S:18S ≥ 1.0, >1 μg. Libraries were sequenced on Illumina Novaseq 6000 (Majorbio) [78].

Differential expression (adjusted *p* < 0.05, |FC| > 1) was analyzed via volcano plots/heatmaps on Majorbio Cloud Platform (cloud.majorbio.com). Functional enrichment (GO/KEGG) was performed using gene clustering. Data are deposited in the National Genomics Data Center (NGDC) GRA (PRJCA048076/CRA031275).

### 4.8. Real-Time RT-PCR

*C. albicans* at 1 × 10^5^ CFU/mL were treated with 60 μg/mL EV or PBS for 6 h, 30 °C, in RPMI 1640. Fungal cells were collected via centrifugation at 4000 rpm for 5 min at 4 °C and were subsequently resuspended in TRIzol reagent (Invitrogen, CA, USA). RNA extraction was performed using a TaKaRa MiniBEST Universal RNA Extraction kit (Takara, Tokyo, Japan) according to the manufacturer’s instructions. Reverse transcription was conducted according to protocols to yield double-stranded cDNA [78,79]. Real-time RT-PCR (RT-qPCR) analysis was then carried out utilizing the primers listed in Table S1. Gene amplification employed a two-step method as reported previously [80].

### 4.9. Selective Depletion of EV Components

Protein Removal: A volume of 60 μL EVs was incubated with 1 μL Proteinase K (APExBIO, Houston, TX, USA) at 37 °C for 1.5 h, followed by inactivation with 1 μL phenylmethylsulfonyl fluoride (APExBIO, Houston, TX, USA) at room temperature for 30 min. Proteinase K was then inactivated by adding 1 mM phenylmethanesulfonyl fluoride (Sigma-Aldrich, St. Louis, MO, USA) and incubating the tube at room temperature for 30 min.

Lipid Removal: A volume of 60 μL EVs was treated with 0.25% sodium deoxycholate (Solarbio, Beijing, China) at 4 °C for 24 h.

Nucleic Acid Removal: Double-stranded DNA—1.2 μL DNase I (Thermo Fisher Scientific, Waltham, MA, USA) was added to 60 μL EVs, followed by incubation at 37 °C for 15 min. Single-stranded DNA/RNA—1.2 μL S1 Nuclease (Thermo Fisher Scientific, Waltham, MA, USA) was mixed with 60 μL EVs and incubated at room temperature for 30 min.

### 4.10. EV Cargo Protein Profiling

EVs were lysed with RIPA buffer (containing 1% protease inhibitor; Thermo Fisher, Waltham, MA, USA) on ice (30 min), followed by centrifugation (12,000× *g*, 4 °C, 15 min). Proteins were quantified via BCA assay (Pierce^TM^ Kit; Thermo Fisher, Waltham, MA, USA). Aliquots (100 μg) were reduced with 10 mM TCEP (37 °C, 60 min), alkylated with 40 mM iodoacetamide (RT, 40 min), and acetone-precipitated. Pellets were dissolved in 100 mM TEAB (Sigma-Aldrich, St. Louis, MO, USA) and digested with trypsin (MS-grade, Promega; 1:50 enzyme/protein, 37 °C, overnight). Peptides were desalted (Oasis^®^ HLB/MCX plates; Waters Corporation, Milford, MA, USA) and analyzed via LC-MS/MS on a timsTOF_HT mass spectrometer (Bruker, Bremen, Germany) coupled to a VanquishNeo UHPLC (Thermo Fisher, Waltham, MA, USA). Separation incorporated a homemade column (15 cm × 100 μm, 1.7 μm) with an 8–99% acetonitrile/0.1% formic acid gradient (300 nL/min, 8 min). Data acquisition was performed using Compass HyStar (Version 5.0, Bruker Daltonics, Billerica, MA, USA) and Xcalibur (Version 4.4, Thermo Fisher Scientific, Waltham, MA, USA). Proteomics data are deposited in ProteomeXchange (PXD063088) via iProX [81,82].

### 4.11. Murine Model of Systemic Infection

The candidemia mouse model was established as previously reported [83]. Female BALB/c mice (6–8 weeks old) with body weights ranging from 18 to 20 g were obtained from the Laboratory Animal Center of Sichuan University (China). The mice were divided into four groups, with a minimum of fifteen mice in each group. Briefly, the mice received intraperitoneal injections of cyclophosphamide (Solarbio, Beijing, China) at a dosage of 100 mg/kg from the first day to the third day. On the 4th day, the mice were stratified into four groups and administered intravenous injections of one of the following solutions: 100 μL (60 μg/mL) EVs or PBS-pretreated *C. albicans* SC5314 (1 × 10^6^ CFU/mL), and 100 μL (60 μg/mL) EVs or PBS-pretreated *nrg1Δ/Δ* mutants (1 × 10^6^ CFU/mL). All groups were pretreated for 24 h, and all strains were centrifuged to remove unbound EVs prior to injection. Mice were closely monitored for the probability of survival in all groups, with daily recordings of time to death and body weight. On the 5th day, six mice from each group were euthanized via ketamine/xylazine overdose, and tissue samples of the liver, brain, and kidneys were harvested. The tissues were longitudinally bisected; one half was immersed in 4% paraformaldehyde (Beyotime, Chengdu, China) for fixation, while the other half was preserved in PBS solution for subsequent analyses. The remaining mice were used to observe survival. Tissue samples were weighed after immersion in PBS solution, then homogenized in a mortar until no visible particles remained. The normalized index is expressed as CFU/tissue weight. After the remaining half of the tissue was used for fixation, it was analyzed via hematoxylin–eosin (HE) and periodic acid–Schiff (PAS) staining.

### 4.12. Statistical Analysis

All experiments were performed in triplicate with ≥3 biological replicates. Data are expressed as mean ± SD. Statistical significance was determined using one-way ANOVA with Student’s *t*-test or Dunnett’s T3 test for parametric data. The Kruskal–Wallis test with the Mann–Whitney U test was used for non-parametric data. Survival analysis was analyzed using the Kaplan–Meier method. Significance thresholds were set at * *p* < 0.05, **** *p* < 0.0001, ns (*p* > 0.05). Analyses were performed using GraphPad Prism 8.3.1.

## 5. Conclusions

In conclusion, *C. albicans* EVs were identified for the first time to repress self-hyphal development and pathogenesis by upregulating *SKO1*, thereby increasing *NRG1* expression. These results indicate new functions of *C. albicans* EVs and provide novel insights into the mechanisms of *C. albicans* infection, commensalism, and potential therapeutic strategies.

## Data Availability

All data and materials are available. All data generated or analyzed during this study are included in this published article. The raw transcriptomic sequencing data generated in this study have been deposited in the National Center for Biotechnology Information (NCBI) database (BioProject accession number: PRJNA877381/CRA031275). The mass spectrometry proteomics data have been deposited in the ProteomeXchange Consortium via the iProX partner repository (dataset identifier: PXD063088).

## Supplementary Materials

The following supporting information can be downloaded at: https://www.mdpi.com/article/10.3390/ijms27010495/s1.
