# Supplementary material for: Candida albicans Extracellular Vesicles Upregulate Nrg1 Transcription Repressor to Inhibit Self-Hyphal Development and Candidemia
Source: Int J Mol Sci. 2026 Jan 3;27(1):495. doi: 10.3390/ijms27010495 (PMC12786599; doi:10.3390/ijms27010495)
Supplement: Supplementary file 1 [file ijms-27-00495-s001.zip › Supplementary Material-Figure S1.pdf]

Candida albicans extracellular vesicles upregulate Nrg1 transcription repressor to inhibit self-hyphal development and candidemia

Yu Wei[1#], Yujie Zhou[3#], Bolei Li[1], Zheng Wang[1], Binyou Liao[1], Jiannan Wang[1], Jingzhi Zhou[1], Yawen Zong[1], Ding Chen[1], Jiawei Shen[1], Yangyang Shi[1], Xuedong Zhou[1], Ga Liao[1], Lichen Gou[1], Zhuoli Zhu[1], Lei Cheng[1*], Biao Ren[1,2*]

Author Affiliation

1. State Key Laboratory of Oral Diseases &National Clinical Research Center for Oral Diseases, West China School of Stomatology, Sichuan University, Chengdu 610000, Sichuan Province, China

2. Tianfu Jiangxi Laboratory, Chengdu 641419, Sichuan Province, China

3. Guangdong Provincial Key Laboratory of Stomatology,Guanghua School of Stomatology, Sun Yat-sen University, Guangzhou, China

# These authors are co-first authors of the article.

*Co-corresponding:

Lei Cheng, State Key Laboratory of Oral Diseases &National Clinical Research Center for Oral Diseases, Sichuan University, Chengdu 610041, China. Email: chenglei@scu.edu.cn

Biao Ren, State Key Laboratory of Oral Diseases &National Clinical Research Center for Oral Diseases, West China School of Stomatology, Sichuan University, Chengdu 610000, Sichuan Province, China. Email: renbiao@scu.edu.cn

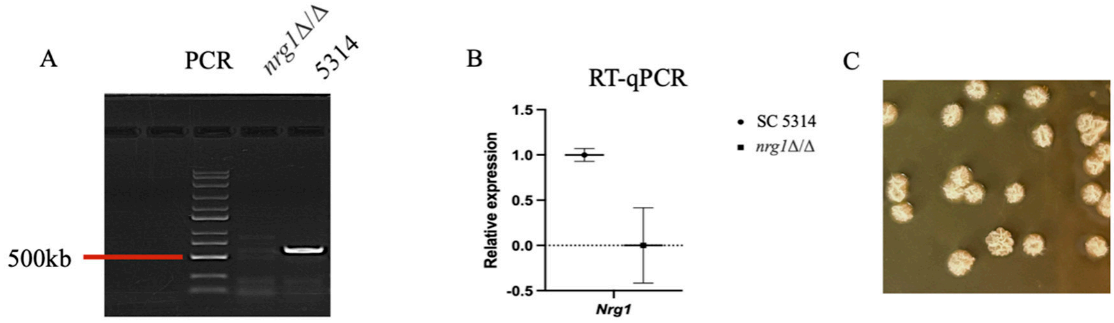

Figure S1 Validation of the *nrg1Δ/Δ* mutant strain. (A) Genomic deletion of the NRG1 fragment was confirmed by PCR. (B) NRG1 transcriptional deficiency was validated by RT-PCR. (C) Colony morphology of the *nrg1Δ/Δ* mutant strain on solid media.
