# Supplementary material for: Candida albicans Extracellular Vesicles Upregulate Nrg1 Transcription Repressor to Inhibit Self-Hyphal Development and Candidemia
Source: Int J Mol Sci. 2026 Jan 3;27(1):495. doi: 10.3390/ijms27010495 (PMC12786599; doi:10.3390/ijms27010495)
Supplement: Supplementary file 1 [file ijms-27-00495-s001.zip › Supplementary Material-Tables.pdf]

Candida albicans extracellular vesicles upregulate Nrg1 transcription repressor to inhibit self-hyphal development and candidemia

Yu Wei[1#], Yujie Zhou[3#], Bolei Li[1], Zheng Wang[1], Binyou Liao[1], Jiannan Wang[1], Jingzhi Zhou[1], Yawen Zong[1], Ding Chen[1], Jiawei Shen[1], Yangyang Shi[1], Xuedong Zhou[1], Ga Liao[1], Lichen Gou[1], Zhuoli Zhu[1], Lei Cheng[1*], Biao Ren[1,2*]

Author Affiliation

1. State Key Laboratory of Oral Diseases &National Clinical Research Center for Oral Diseases, West China School of Stomatology, Sichuan University, Chengdu 610000, Sichuan Province, China

2. Tianfu Jiangxi Laboratory, Chengdu 641419, Sichuan Province, China

3. Guangdong Provincial Key Laboratory of Stomatology,Guanghua School of Stomatology, Sun Yat-sen University, Guangzhou, China

# These authors are co-first authors of the article.

*Co-corresponding:

Lei Cheng, State Key Laboratory of Oral Diseases &National Clinical Research Center for Oral Diseases, Sichuan University, Chengdu 610041, China. Email: chenglei@scu.edu.cn

Biao Ren, State Key Laboratory of Oral Diseases &National Clinical Research Center for Oral Diseases, West China School of Stomatology, Sichuan University, Chengdu 610000, Sichuan Province, China. Email: renbiao@scu.edu.cn

Table S1 Primer sequences used in RT-qPCR

Primers	Sequence
18S-F	CTAGGGATCGGTTGTTGTTCT
18S-R	TTGTGTCTGGACCTGGTGAGT
NRG1-F	AACGTCACCAACAACCCATG
NRG1-R	TCGAACACACTTTGCAGACG
SKO1-F	GACGCCATTTCCGATGTCAA
SKO1-R	CCAGGTGTGAACAAAGGTGG
RBF1-F	CGCCTCAATTGCTGCTGTTA
RBF1-R	AAAACCGGCACCACTTGATG
TUP1-F	TGACGAGTCCTCCAACGAAA
TUP1-R	AAATGTCTTGTTCGTGGCCC
RAS2-F	CGTGCAAACGTAATGGGAGT
RAS2-R	GAAGTTTGCGATTGGGTGGT
MEP2-F	TGATTCCTGGTGTGGGTCTC
MEP2-R	GGTGCACCCAAAACATCCTT
IHD1-F	CCAACAACAATGGCTCCACT
IHD1-R	CAGTGGATGCGGTATTGGTG
IHD2-F	ACGACAACAACTCCTCCTGA
IHD2-R	ACCTCGTCAAAATCTCTCCCT
QCR8-F	GGTTGTTTCGCCTTGTGACT
QCR8-R	GCACTTGCGGTATACTTGGG
SHA3-F	GCACCAGAAAGAACCACCAA
SHA3-R	AATGGGCCATGGGTTTCTTG
BRG1-F	CAGCCACAATTACCTCCTGC
BRG1-R	GCGTATTGTAATGGGGCAGG
CSR1-F	ACTTCGGTGAAAATCGTGCC
CSR1-R	CCCACGCCAACAACAATGAT
HYR1-F	ACGAAGGTTCAGGTCAAGGT
HYR1-R	GAACCAGAGTGTGAACCTGC
HWP1-F	TCCGGAATCTAGTGCTGTCG
HWP1-R	GCAGCACCGAAAGTCAATCT
UME6-F	GGTGGTGTCAGTGTTAGTGC
UME6-R	AGATGTTGGTGGTGGTGGAA
ALS3-F	CTACCGCTGTGACCACCTTA
ALS3-R	CAGTTTCCCCAATTGGTGCA

***SAP5*-F**	**GGGGACGAAATGTTGATGCT**
***SAP5*-R**	**TTCGGAAACAGGAACGGAGA**
***ECE1*-F**	**TTTGATGGCGTCCTGGAAAC**
***ECE1*-R**	**TACTGAGCCGGCATCTCTTT**
***YWP1*-F**	**ATCGGGTTCTGCTTCTCCAT**
***YWP1*-R**	**CAGTGGTAGCATGACAAGCA**

Table S2 *C. albicans* cargo proteins associated with hyphae developments

Accession	Symbol	Protein name	Description	GO_term	Domain	Domain description
A0A1D8PDC4	ATP1	ATP synthase subunit alpha	A0A1D8PDC4_CANAL ATP synthase subunit alpha OS=Candida albicans (strain SC5314 / ATCC MYA-2876) OX=237561 GN=ATP1 PE=3 SV=1	BP:nucleotide biosynthetic process	ATP-synt_ab;;ATP-synt_ab_C;;ATP-synt_ab_N	ATP synthase alpha/beta family, nucleotide-binding domain;;ATP synthase alpha/beta chain, C terminal domain;;ATP synthase alpha/beta family, beta-barrel domain
A0A1D8PDL7	CAALFM_C105630CA	Importin N-terminal domain-containing protein	A0A1D8PDL7_CANAL Importin N-terminal domain-containing protein OS=Candida albicans (strain SC5314 / ATCC MYA-2876) OX=237561 GN=CAALFM_C105630CA PE=4 SV=1	BP:protein localization to nucleus	HEAT_EZ;;HEAT	HEAT-like repeat;;HEAT repeat
A0A1D8PDT3	RPS14B	Small ribosomal subunit protein uS11	RS14B_CANAL Small ribosomal subunit protein uS11 OS=Candida albicans (strain SC5314 /	BP:cellular component assembly	Ribosomal_S11	Ribosomal protein S11

			ATCC MYA-2876) OX=237561 GN=RPS14B PE=1 SV=1			
A0A1D8PEB1	PLB5	Lysophospholipase	A0A1D8PEB1_CANAL Lysophospholipase OS=Candida albicans (strain SC5314 / ATCC MYA-2876) OX=237561 GN=PLB5 PE=3 SV=1	BP:organophosphate catabolic process	PLA2_B	Lysophospholipase catalytic domain
A0A1D8PF68	SAM2	S-adenosylmethionine synthase	A0A1D8PF68_CANAL S-adenosylmethionine synthase OS=Candida albicans (strain SC5314 / ATCC MYA-2876) OX=237561 GN=SAM2 PE=3 SV=1	BP:one-carbon metabolic process	S-AdoMet_synt_C;;S-AdoMet_synt_M;;S-AdoMet_synt_N	S-adenosylmethionine synthetase, C-terminal domain;;S-adenosylmethionine synthetase, central domain;;S-adenosylmethionine synthetase, N-terminal domain
A0A1D8PF97	CAALFM_C111930WA	PH domain-containing protein	A0A1D8PF97_CANAL PH domain-containing protein OS=Candida albicans (strain SC5314 / ATCC MYA-2876)	BP:filamentous growth	PH	PH domain

			OX=237561 GN=CAALFM_C111930 WA PE=4 SV=1			
A0A1D8PFL9	RPL14	Large ribosomal subunit protein eL14	RL14_CANAL Large ribosomal subunit protein eL14 OS=Candida albicans (strain SC5314 / ATCC MYA-2876) OX=237561 GN=RPL14 PE=1 SV=1	BP:nitrogen compound metabolic process	Ribosomal_L14e	Ribosomal protein L14
A0A1D8PGB7	ENT2	Epsin	A0A1D8PGB7_CANAL Epsin OS=Candida albicans (strain SC5314 / ATCC MYA-2876) OX=237561 GN=ENT2 PE=1 SV=1	BP:hyphal growth	ENTH	ENTH domain
A0A1D8PH31	TAF14	TATA-binding protein-associated factor	A0A1D8PH31_CANAL TATA-binding protein-associated factor OS=Candida albicans (strain SC5314 / ATCC MYA-2876) OX=237561 GN=TAF14 PE=4 SV=1	BP:regulation of nucleobase-containing compound metabolic process	YEATS;;pYEATS;;BET	YEATS family;;prokaryotic YEATS domain;;Bromodomain extra-terminal - transcription regulation
A0A1D8PJG1	GPA2	Guanine nucleotide-binding protein subunit alpha	A0A1D8PJG1_CANAL Guanine nucleotide-binding protein subunit	BP:cellular response to neutral pH	G-alpha;;Arf	G-protein alpha subunit;;ADP-

			alpha OS=Candida albicans (strain SC5314 / ATCC MYA-2876) OX=237561 GN=GPA2 PE=4 SV=1			ribosylation factor family
A0A1D8PKB4	YCK2	Serine/threonine protein kinase	A0A1D8PKB4_CANAL non-specific serine/threonine protein kinase OS=Candida albicans (strain SC5314 / ATCC MYA-2876) OX=237561 GN=YCK2 PE=1 SV=1	BP:regulation of cell wall organization or biogenesis	Pkinase;;PK_Tyr_Ser-Thr;;Pkinase_fungal	Protein kinase domain;;Protein tyrosine and serine/threonine kinase;;Fungal protein kinase
A0A1D8PP43	ADH1	Adh1p	A0A1D8PP43_CANAL alcohol dehydrogenase OS=Candida albicans (strain SC5314 / ATCC MYA-2876) OX=237561 GN=ADH1 PE=3 SV=1	BP:response to biotic stimulus	ADH_zinc_N;;ADH_N;;ADH_zinc_N_2	Zinc-binding dehydrogenase;;Alcohol dehydrogenase GroES-like domain;;Zinc-binding dehydrogenase
A0A1D8PPT5	RPL23A	Large ribosomal subunit protein uL14	RL23A_CANAL Large ribosomal subunit protein uL14 OS=Candida albicans (strain SC5314 / ATCC MYA-2876)	BP:cellular biosynthetic process	Ribosomal_L14	Ribosomal protein L14p/L23e

			OX=237561 GN=RPL23A PE=1 SV=1			
A0A1D8PQQ3	GPH1	Alpha-1,4 glucan phosphorylase	A0A1D8PQQ3_CANAL Alpha-1,4 glucan phosphorylase OS=Candida albicans (strain SC5314 / ATCC MYA-2876) OX=237561 GN=GPH1 PE=3 SV=1	BP:glucan catabolic process	Phosphorylase	Carbohydrate phosphorylase
A0A1D8PS71	PHO112	acid phosphatase	PHYA_CANAL Phytase PHO112 OS=Candida albicans (strain SC5314 / ATCC MYA-2876) OX=237561 GN=PHO112 PE=1 SV=1	BP:hyphal growth	His_Phos_2	Histidine phosphatase superfamily
A0A1D8PSV5	RPS3	Small ribosomal subunit protein uS3	RS3_CANAL Small ribosomal subunit protein uS3 OS=Candida albicans (strain SC5314 / ATCC MYA-2876) OX=237561 GN=RPS3 PE=1 SV=1	BP:nucleobase-containing compound transport	Ribosomal_S3_C;;KH_2	Ribosomal protein S3, C-terminal domain;;KH domain

A0A1D8PUB4	XYL2	L-iditol 2-dehydrogenase	A0A1D8PUB4_CANAL L-iditol 2-dehydrogenase OS=Candida albicans (strain SC5314 / ATCC MYA-2876) OX=237561 GN=XYL2 PE=3 SV=1	BP:alditol catabolic process	ADH_N;;ADH_zinc_N;;Glu_dehyd_C	Alcohol dehydrogenase GroES-like domain;;Zinc-binding dehydrogenase;;Glucose dehydrogenase C-terminus
A0A8H6BST6	FOB64_006937	Ribosomal protein L37a	A0A8H6BST6_CANAX Ribosomal protein L37a OS=Candida albicans OX=5476 GN=FOB64_006937 PE=3 SV=1	BP:cellular response to carbon dioxide	Ribosomal_L37ae;;F-box-like	Ribosomal L37ae protein family;;F-box-like
A0A8H6BTE2	FOB64_005018	YEATS family protein	A0A8H6BTE2_CANAX YEATS family protein OS=Candida albicans OX=5476 GN=FOB64_005018 PE=4 SV=1	BP:regulation of nucleobase-containing compound metabolic process	YEATS;;pYEATS;;BET	YEATS family;;prokaryotic YEATS domain;;Bromodomain extra-terminal - transcription regulation
A0A8H6BTY1	FOB64_004058	KilA-N domain family protein	A0A8H6BTY1_CANAX KilA-N domain family protein OS=Candida albicans OX=5476	BP:regulation of primary metabolic process	KilA-N	KilA-N domain

			GN=FOB64_004058 PE=3 SV=1			
A0A8H6BUQ8	FOB64_005740	Hyphally-regulated cell wall protein N-terminal domain-containing protein	A0A8H6BUQ8_CANAX Hyphally-regulated cell wall protein N-terminal domain-containing protein OS=Candida albicans OX=5476 GN=FOB64_005740 PE=4 SV=1			
A0A8H6BWB2	MNS1	alpha-1,2-Mannosidase	A0A8H6BWB2_CANAX alpha-1,2-Mannosidase OS=Candida albicans OX=5476 GN=MNS1 PE=3 SV=1	BP:response to biotic stimulus	Glyco_hydro_47	Glycosyl hydrolase family 47
A0A8H6BWS8	FOB64_005402	Elongation factor 1-alpha	A0A8H6BWS8_CANAX Elongation factor 1-alpha OS=Candida albicans OX=5476 GN=FOB64_005402 PE=3 SV=1	BP:metabolic process	GTP_EFTU;;GTP_EFTU_D3;;GTP_EFTU_D2	Elongation factor Tu GTP binding domain;;Elongation factor Tu C-terminal domain;;Elongation factor Tu domain 2
A0A8H6BXH3	FOB64_003763	NDT80 / PhoG like DNA-binding family protein	A0A8H6BXH3_CANAX NDT80 / PhoG like DNA-binding family protein OS=Candida albicans OX=5476 GN=FOB64_003763 PE=4 SV=1	BP:regulation of primary metabolic process	NDT80_PhoG	NDT80 / PhoG like DNA-binding family

A0A8H6BY53	HYR1	Hyphally regulated cell wall protein 1	A0A8H6BY53_CANAX Hyphally regulated cell wall protein 1 OS=Candida albicans OX=5476 GN=HYR1 PE=4 SV=1			
A0A8H6BZ50	FOB64_003021	PH domain-containing protein	A0A8H6BZ50_CANAX PH domain-containing protein OS=Candida albicans OX=5476 GN=FOB64_003021 PE=4 SV=1	BP:filamentous growth	PH	PH domain
A0A8H6BZJ9	FOB64_004579	acid phosphatase	A0A8H6BZJ9_CANAX Histidine phosphatase (Branch 2) family protein OS=Candida albicans OX=5476 GN=FOB64_004579 PE=3 SV=1	BP:hyphal growth	His_Phos_2	Histidine phosphatase superfamily
A0A8H6C1G8	FOB64_003142	nicotinamide-nucleotide adenylyltransferase	A0A8H6C1G8_CANAX Nicotinamide-nucleotide adenylyltransferase OS=Candida albicans OX=5476 GN=FOB64_003142 PE=4 SV=1	BP:NAD biosynthesis via nicotinamide riboside salvage pathway	------	------
A0A8H6C1I5	FOB64_001426	Pyruvate dehydrogenase E1 component subunit alpha	A0A8H6C1I5_CANAX Pyruvate dehydrogenase E1 component subunit alpha OS=Candida	BP:nucleotide biosynthetic process	E1_dh	Dehydrogenase E1 component

			albicans OX=5476 GN=FOB64_001426 PE=4 SV=1			
A0A8H6C300	FOB64_000497	DJ-1/PfpI family protein	A0A8H6C300_CANAX D-lactate dehydratase OS=Candida albicans OX=5476 GN=FOB64_000497 PE=3 SV=1	BP:carboxylic acid metabolic process	DJ-1_PfpI	DJ-1/PfpI family
A0A8H6C375	FOB64_000203	Protein kinase domain family protein	A0A8H6C375_CANAX non-specific serine/threonine protein kinase OS=Candida albicans OX=5476 GN=FOB64_000203 PE=3 SV=1	BP:regulation of cell wall organization or biogenesis	Pkinase;;PK_Tyr_Ser-Thr;;Pkinase_fungal	Protein kinase domain;;Protein tyrosine and serine/threonine kinase;;Fungal protein kinase
A0A8H6C3Q1	FOB64_000540	G-protein alpha subunit family protein	A0A8H6C3Q1_CANAX G-protein alpha subunit family protein OS=Candida albicans OX=5476 GN=FOB64_000540 PE=4 SV=1	BP:cellular response to neutral pH	G-alpha;;Arf	G-protein alpha subunit;;ADP-ribosylation factor family
A0A8H6F0P8	FOB64_005926	Lysophospholipase	A0A8H6F0P8_CANAX Lysophospholipase OS=Candida albicans	BP:organophosphate	PLA2_B;;His_Phos_1	Lysophospholipase catalytic domain;;Histidine

			OX=5476 GN=FOB64_005926 PE=3 SV=1	catabolic process		phosphatase superfamily
A0A8H6F163	FOB64_004737	ENTH domain family protein	A0A8H6F163_CANAX ENTH domain family protein OS=Candida albicans OX=5476 GN=FOB64_004737 PE=3 SV=1	BP:hyphal growth	ENTH	ENTH domain
A0A8H6F2W4	FOB64_003329	PH domain-containing protein	A0A8H6F2W4_CANAX PH domain-containing protein OS=Candida albicans OX=5476 GN=FOB64_003329 PE=4 SV=1	BP:filamentous growth	PH	PH domain
A0A8H6F309	CDC28	Cyclin-dependent kinase 1	A0A8H6F309_CANAX Cyclin-dependent kinase 1 OS=Candida albicans OX=5476 GN=CDC28 PE=3 SV=1	BP:protein localization to nuclear periphery	Pkinase;;PK_Tyr_Ser-Thr	Protein kinase domain;;Protein tyrosine and serine/threonine kinase
A0A8H6F530	SSA4	Heat shock protein SSA4	A0A8H6F530_CANAX Heat shock protein SSA4 OS=Candida albicans OX=5476 GN=SSA4 PE=3 SV=1	BP:protein localization	HSP70;;MreB_Mbl	Hsp70 protein;;MreB/Mbl protein

A0AB34PS73	-	MG3_03041	A0AB34PS73_CANAX Casein kinase 1 OS=Candida albicans P78048 OX=1094989 GN=MG3_03041 PE=4 SV=1	BP:regulation of cell wall organization or biogenesis	Pkinase;;PK_Tyr_Ser-Thr;;Pkinase_fungal	Protein kinase domain;;Protein tyrosine and serine/threonine kinase;;Fungal protein kinase
A0AB34PTQ9	-	MG3_02697	A0AB34PTQ9_CANAX Guanine nucleotide-binding protein subunit alpha, other OS=Candida albicans P78048 OX=1094989 GN=MG3_02697 PE=4 SV=1	BP:cellular response to neutral pH	G-alpha;;Arf	G-protein alpha subunit;;ADP-ribosylation factor family
A0AB34PWW4	-	MG3_01835	A0AB34PWW4_CANAX Transcription initiation factor TFIID/TFIIF subunit OS=Candida albicans P78048 OX=1094989 GN=MG3_01835 PE=4 SV=1	BP:regulation of nucleobase-containing compound metabolic process	YEATS;;pYEATS;;BET	YEATS family;;prokaryotic YEATS domain;;Bromodomain extra-terminal - transcription regulation
A0AB34PXU0	-	MG3_01601	A0AB34PXU0_CANAX Epsin OS=Candida albicans P78048 OX=1094989	BP:hyphal growth	ENTH	ENTH domain

			GN=MG3_01601 PE=4 SV=1			
A0AB34PYU8	-	MG3_00840	A0AB34PYU8_CANAX Lysophospholipase 3 OS=Candida albicans P78048 OX=1094989 GN=MG3_00840 PE=4 SV=1	BP:organophosphate catabolic process	PLA2_B	Lysophospholipase catalytic domain
A0AB34PZ64	-	MG3_00405	A0AB34PZ64_CANAX Mannosyl-oligosaccharide 1,2-alpha-mannosidase OS=Candida albicans P78048 OX=1094989 GN=MG3_00405 PE=4 SV=1	BP:response to biotic stimulus	Glyco_hydro_47	Glycosyl hydrolase family 47
A0AB34PZ64	-	MG3_00405	A0AB34PZ64_CANAX Mannosyl-oligosaccharide 1,2-alpha-mannosidase OS=Candida albicans P78048 OX=1094989 GN=MG3_00405 PE=4 SV=1	BP:response to biotic stimulus	Glyco_hydro_47	Glycosyl hydrolase family 47

A0AB34PZP3	-	MG3_00590	A0AB34PZP3_CANAX Uncharacterized protein OS=Candida albicans P78048 OX=1094989 GN=MG3_00590 PE=4 SV=1	BP:protein localization to nucleus	HEAT_EZ;;HEAT		HEAT-like repeat;;HEAT repeat
A0AB34PZS9	-	MG3_01205	A0AB34PZS9_CANAX CCR4-NOT transcriptional complex subunit CAF120 OS=Candida albicans P78048 OX=1094989 GN=MG3_01205 PE=4 SV=1	BP:filamentous growth	PH		PH domain
A0AB34Q0A5	-	MG3_00460	A0AB34Q0A5_CANAX Hsp72-like protein OS=Candida albicans P78048 OX=1094989 GN=MG3_00460 PE=4 SV=1	BP:chaperone-mediated protein folding	HSP70;;MreB_Mbl		Hsp70 protein;;MreB/Mbl protein
A0AB34Q1X2	-	MG3_01353	A0AB34Q1X2_CANAX Hyphally-regulated protein OS=Candida albicans P78048 OX=1094989 GN=MG3_01353 PE=4 SV=1				
C4YCL4	CAWG_00248	PH domain-containing protein	C4YCL4_CANAW PH domain-containing protein OS=Candida albicans (strain WO-1)	BP:filamentous growth	PH		PH domain

			OX=294748 GN=CAWG_00248 PE=4 SV=1			
C4YCQ7	CAWG_00291	S-adenosylmethionine synthase	C4YCQ7_CANAW S-adenosylmethionine synthase OS=Candida albicans (strain WO-1) OX=294748 GN=CAWG_00291 PE=3 SV=1	BP:one-carbon metabolic process	S-AdoMet_synt_C;;S-AdoMet_synt_M;;S-AdoMet_synt_N	S-adenosylmethionine synthetase, C-terminal domain;;S-adenosylmethionine synthetase, central domain;;S-adenosylmethionine synthetase, N-terminal domain
C4YDK3	CAWG_00600	Lysophospholipase	C4YDK3_CANAW Lysophospholipase OS=Candida albicans (strain WO-1) OX=294748 GN=CAWG_00600 PE=3 SV=1	BP:organophosphate catabolic process	PLA2_B	Lysophospholipase catalytic domain
C4YER0	CAWG_01018	alpha-1,2-Mannosidase	C4YER0_CANAW alpha-1,2-Mannosidase OS=Candida albicans (strain WO-1) OX=294748	BP:response to biotic stimulus	Glyco_hydro_47	Glycosyl hydrolase family 47

			GN=CAWG_01018 PE=3 SV=1			
C4YFX2	TUP1	Transcriptional repressor TUP1	TUP1_CANAW Transcriptional repressor TUP1 OS=Candida albicans (strain WO-1) OX=294748 GN=TUP1 PE=4 SV=1	BP:detection of biotic stimulus	WD40;;Tup_N;;NBCH_WD40;;ANAPC4_WD40;;RMC1_N;;Cytochrom_D1	WD domain, G-beta repeat;;Tup N-terminal;;Neurobeachin beta propeller domain;;Anaphase-promoting complex subunit 4 WD40 domain;;Regulator of MON1-CCZ1 complex, N-terminal;;Cytochrome D1 heme domain
C4YFZ1	CAWG_00103	Heat shock protein SSA4	C4YFZ1_CANAW Heat shock protein SSA4 OS=Candida albicans (strain WO-1) OX=294748 GN=CAWG_00103 PE=3 SV=1	BP:protein localization	HSP70;;MreB_Mbl	Hsp70 protein;;MreB/Mbl protein
C4YIA6	CAWG_04178	Transcription initiation factor TFIID subunit 14	C4YIA6_CANAW Transcription initiation factor TFIID subunit 14 OS=Candida albicans	BP:regulation of nucleobase-containing	YEATS;;pYEATS;;BET	YEATS family;;prokaryotic YEATS domain;;Bromodo

			(strain WO-1) OX=294748 GN=CAWG_04178 PE=4 SV=1	compound metabolic process		main extra-terminal - transcription regulation
C4YIL8	CAWG_04294	Pyruvate kinase	C4YIL8_CANAW Pyruvate kinase OS=Candida albicans (strain WO-1) OX=294748 GN=CAWG_04294 PE=3 SV=1	BP:positive regulation of response to stimulus	PK;;PK_C	Pyruvate kinase, barrel domain;;Pyruvate kinase, alpha/beta domain
C4YIY2	CAWG_03794	NDT80 domain-containing protein	C4YIY2_CANAW NDT80 domain-containing protein OS=Candida albicans (strain WO-1) OX=294748 GN=CAWG_03794 PE=4 SV=1	BP:regulation of primary metabolic process	NDT80_PhoG	NDT80 / PhoG like DNA-binding family
C4YJ99	CAWG_03912	ENTH domain-containing protein	C4YJ99_CANAW ENTH domain-containing protein OS=Candida albicans (strain WO-1) OX=294748	BP:hyphal growth	ENTH	ENTH domain

			GN=CAWG_03912 PE=3 SV=1			
C4YKT4	RAS1	Ras-like protein 1	RAS1_CANAW Ras-like protein 1 OS=Candida albicans (strain WO-1) OX=294748 GN=RAS1 PE=3 SV=1	BP:regulation of protein localization	Ras;;Roc;;Arf;;GTP_EFTU	Ras family;;Ras of Complex, Roc, domain of DAPkinase;;ADP-ribosylation factor family;;Elongation factor Tu GTP binding domain
C4YLU5	CAWG_01814	40S ribosomal protein S3	C4YLU5_CANAW 40S ribosomal protein S3 OS=Candida albicans (strain WO-1) OX=294748 GN=CAWG_01814 PE=3 SV=1	BP:nucleobase-containing compound transport	Ribosomal_S3_C;;KH_2	Ribosomal protein S3, C-terminal domain;;KH domain
C4YMK1	CAWG_02083	HTH APSES-type domain-containing protein	C4YMK1_CANAW HTH APSES-type domain-containing protein OS=Candida albicans (strain WO-1) OX=294748 GN=CAWG_02083 PE=3 SV=1	BP:regulation of primary metabolic process	KilA-N	KilA-N domain

C4YNU2	CAWG_02875	Casein kinase I isoform gamma-1	C4YNU2_CANAW non-specific serine/threonine protein kinase OS=Candida albicans (strain WO-1) OX=294748 GN=CAWG_02875 PE=3 SV=1	BP:regulation of cell wall organization or biogenesis	Pkinase;;PK_Tyr_Ser-Thr;;Pkinase_fungal	Protein kinase domain;;Protein tyrosine and serine/threonine kinase;;Fungal protein kinase
C4YPJ2	CAWG_02393	Hyphally-regulated cell wall protein N-terminal domain-containing protein	C4YPJ2_CANAW Hyphally-regulated cell wall protein N-terminal domain-containing protein (Fragment) OS=Candida albicans (strain WO-1) OX=294748 GN=CAWG_02393 PE=4 SV=1			
C4YPJ4	CAWG_02395	Hyphally-regulated cell wall protein N-terminal domain-containing protein	C4YPJ4_CANAW Hyphally-regulated cell wall protein N-terminal domain-containing protein OS=Candida albicans (strain WO-1) OX=294748 GN=CAWG_02395 PE=4 SV=1			
C4YPZ6	CAWG_02550	Guanine nucleotide-binding protein alpha-2 subunit	C4YPZ6_CANAW Guanine nucleotide-binding protein alpha-2 subunit OS=Candida albicans (strain WO-1) OX=294748 GN=CAWG_02550 PE=4 SV=1	BP:cellular response to neutral pH	G-alpha;;Arf	G-protein alpha subunit;;ADP-ribosylation factor family
C4YQW8	CAWG_04465	Hyphally-regulated cell wall protein N-terminal	C4YQW8_CANAW Hyphally-regulated cell wall protein N-terminal domain-containing protein OS=Candida albicans (strain WO-1) OX=294748 GN=CAWG_04465 PE=4 SV=1			

		domain-containing protein				
C4YS13	CAWG_04871	Alcohol dehydrogenase I	C4YS13_CANAW alcohol dehydrogenase OS=Candida albicans (strain WO-1) OX=294748 GN=CAWG_04871 PE=3 SV=1	BP:response to biotic stimulus	ADH_zinc_N;;ADH_N;;ADH_zinc_N_2	Zinc-binding dehydrogenase;;Alcohol dehydrogenase GroES-like domain;;Zinc-binding dehydrogenase
G9BX82	hsp70	Heat shock protein 70	G9BX82_CANAX Heat shock protein 70 OS=Candida albicans OX=5476 GN=hsp70 PE=3 SV=1	BP:protein localization	HSP70;;MreB_Mbl	Hsp70 protein;;MreB/Mbl protein
O42825	RHO1	GTP-binding protein RHO1	RHO1_CANAL GTP-binding protein RHO1 OS=Candida albicans (strain SC5314 / ATCC MYA-2876) OX=237561 GN=RHO1 PE=3 SV=1	BP:cellular biosynthetic process	Ras;;Roc;;Arf	Ras family;;Ras of Complex, Roc, domain of DAPkinase;;ADP-ribosylation factor family
O94038	ADH2	Alcohol dehydrogenase 2	ADH2_CANAL Alcohol dehydrogenase 2 OS=Candida albicans (strain SC5314 / ATCC	BP:single-species submerged biofilm formation	ADH_N;;ADH_zinc_N;;ADH_zinc_N_2	Alcohol dehydrogenase GroES-like domain;;Zinc-binding

			MYA-2876) OX=237561 GN=ADH2 PE=3 SV=1			dehydrogenase;;Zinc-binding dehydrogenase
P0CY33	CDC42	Cell division control protein 42 homolog	CDC42_CANAL Cell division control protein 42 homolog OS=Candida albicans (strain SC5314 / ATCC MYA-2876) OX=237561 GN=CDC42 PE=3 SV=1	BP:regulation of secretion	Ras;;Roc;;Arf	Ras family;;Ras of Complex, Roc, domain of DAPkinase;;ADP-ribosylation factor family
P30575	ENO1	Enolase 1	ENO1_CANAL Enolase 1 OS=Candida albicans (strain SC5314 / ATCC MYA-2876) OX=237561 GN=ENO1 PE=1 SV=1	BP:negative regulation of response to stimulus	Enolase_C;;Enolase_N	Enolase, C-terminal TIM barrel domain;;Enolase, N-terminal domain
P40910	RPS1	Small ribosomal subunit protein eS1	RS3A_CANAL Small ribosomal subunit protein eS1 OS=Candida albicans (strain SC5314 / ATCC MYA-2876) OX=237561 GN=RPS1 PE=1 SV=3	BP:cellular biosynthetic process	Ribosomal_S3Ae	Ribosomal S3Ae family
P41797	SSA1	Heat shock protein SSA1	HSP71_CANAL Heat shock protein HSP70 OS=Candida albicans (strain SC5314 / ATCC	BP:protein localization	HSP70;;MreB_Mbl	Hsp70 protein;;MreB/Mbl protein

			MYA-2876) OX=237561 GN=HSP70 PE=1 SV=2			
P42800	INO1	Inositol-3-phosphate synthase	INO1_CANAL Inositol-3-phosphate synthase OS=Candida albicans (strain SC5314 / ATCC MYA-2876) OX=237561 GN=INO1 PE=3 SV=1	BP:polyol metabolic process	NAD_binding_5;;Inos-1-P_synth	Myo-inositol-1-phosphate synthase;;Myo-inositol-1-phosphate synthase
P46273	PGK1	Phosphoglycerate kinase	PGK_CANAL Phosphoglycerate kinase OS=Candida albicans (strain SC5314 / ATCC MYA-2876) OX=237561 GN=PGK1 PE=3 SV=1	BP:positive regulation of response to stimulus	PGK	Phosphoglycerate kinase
P46587	SSA2	Heat shock protein SSA2	HSP72_CANAL Heat shock protein SSA2 OS=Candida albicans (strain SC5314 / ATCC MYA-2876) OX=237561 GN=SSA2 PE=1 SV=4	BP:chaperone-mediated protein folding	HSP70;;MreB_Mbl	Hsp70 protein;;MreB/Mbl protein
P46598	HSP90	Heat shock protein 90 homolog	HSP90_CANAL Heat shock protein 90 homolog OS=Candida albicans (strain SC5314 / ATCC MYA-2876)	BP:negative regulation of filamentous growth of a population of	HSP90;;HATPase_c_3;;HATPase_c	Hsp90 protein;;Histidine kinase-, DNA gyrase B-, and HSP90-like ATPase;;Histidine

			OX=237561 GN=HSP90 PE=1 SV=1	unicellular organisms			kinase-, DNA gyrase B-, and HSP90-like ATPase
P46614	CDC19	Pyruvate kinase	KPYK_CANAL Pyruvate kinase OS=Candida albicans (strain SC5314 / ATCC MYA-2876) OX=237561 GN=CDC19 PE=1 SV=3	BP:positive regulation of response to stimulus	PK;;PK_C		Pyruvate kinase, barrel domain;;Pyruvate kinase, alpha/beta domain
P82610	MET6	5-methyltetrahydropteroyltriglutamate--homocysteine methyltransferase	METE_CANAL 5-methyltetrahydropteroyltriglutamate--homocysteine methyltransferase OS=Candida albicans (strain SC5314 / ATCC MYA-2876) OX=237561 GN=MET6 PE=1 SV=2	BP:cellular response to heat	Meth_synt_2;;Meth_synt_1		Cobalamin-independent synthase, Catalytic domain;;Cobalamin-independent synthase, N-terminal domain
P82612	GPM1	Phosphoglycerate mutase	PMGY_CANAL Phosphoglycerate mutase OS=Candida albicans (strain SC5314 / ATCC MYA-2876) OX=237561 GN=GPM1 PE=1 SV=3	BP:monosaccharide biosynthetic process	His_Phos_1		Histidine phosphatase superfamily
P83777	IPP1	Inorganic pyrophosphatase	IPYR_CANAL Inorganic pyrophosphatase	BP:nucleic acid	Pyrophosphatase		Inorganic pyrophosphatase

			OS=Candida albicans (strain SC5314 / ATCC MYA-2876) OX=237561 GN=IPP1 PE=1 SV=3	metabolic process		
P83779	PDC11	Pyruvate decarboxylase	PDC1_CANAL Pyruvate decarboxylase OS=Candida albicans (strain SC5314 / ATCC MYA-2876) OX=237561 GN=PDC11 PE=1 SV=2	BP:carboxylic acid metabolic process	TPP_enzyme_M;;TPP_enzyme_N;;TPP_enzyme_C	Thiamine pyrophosphate enzyme, central domain;;Thiamine pyrophosphate enzyme, N-terminal TPP binding domain;;Thiamine pyrophosphate enzyme, C-terminal TPP binding domain
Q3MPN9	CaJ7.0111	Alpha-1,4 glucan phosphorylase	Q3MPN9_CANAX Alpha-1,4 glucan phosphorylase OS=Candida albicans OX=5476 GN=CaJ7.0111 PE=3 SV=1	BP:glucan catabolic process	Phosphorylase	Carbohydrate phosphorylase

Q59QD6	TEF2	Elongation factor 1-alpha 2	EF1A2_CANAL Elongation factor 1-alpha 2 OS=Candida albicans (strain SC5314 / ATCC MYA-2876) OX=237561 GN=TEF2 PE=3 SV=2	BP:cellular biosynthetic process	GTP_EFTU;;GTP_EFTU_D3;;GTP_EFTU_D2	Elongation factor Tu GTP binding domain;;Elongation factor Tu C-terminal domain;;Elongation factor Tu domain 2
Q59RH5	HAT2	Histone acetyltransferase type B subunit 2	HAT2_CANAL Histone acetyltransferase type B subunit 2 OS=Candida albicans (strain SC5314 / ATCC MYA-2876) OX=237561 GN=HAT2 PE=3 SV=1	BP:negative regulation of filamentous growth of a population of unicellular organisms	CAF1C_H4-bd;;WD40;;ANAPC4_WD40	Histone-binding protein RBBP4 or subunit C of CAF1 complex;;WD domain, G-beta repeat;;Anaphase-promoting complex subunit 4 WD40 domain
Q59X67	EFG1	Enhanced filamentous growth protein 1	EFG1_CANAL Enhanced filamentous growth protein 1 OS=Candida albicans (strain SC5314 / ATCC MYA-2876) OX=237561 GN=EFG1 PE=1 SV=2	BP:regulation of primary metabolic process	KilA-N	KilA-N domain
Q59Y37	PST2	Pst2p	PST2_CANAL NAD(P)H quinone	BP:cellular response to	FMN_red;;Flavodoxin_1	NADPH-dependent FMN

			oxidoreductase PST2 OS=Candida albicans (strain SC5314 / ATCC MYA-2876) OX=237561 GN=PST2 PE=2 SV=1	chemical stimulus		reductase;;Flavodoxin
Q59YF0	SSO2	Protein transport protein SSO2	SSO2_CANAL Protein transport protein SSO2 OS=Candida albicans (strain SC5314 / ATCC MYA-2876) OX=237561 GN=SSO2 PE=3 SV=1	BP:protein localizatio	Syntaxin;;SNARE	Syntaxin;;SNARE domain
Q5A017	TAL1	Transaldolase	Q5A017_CANAL Transaldolase OS=Candida albicans (strain SC5314 / ATCC MYA-2876) OX=237561 GN=TAL1 PE=3 SV=1	BP:NADP metabolic process	TAL_FSA	Transaldolase/Fructose-6-phosphate aldolase
Q5A389	RPS20	Small ribosomal subunit protein uS10	RS20_CANAL Small ribosomal subunit protein uS10 OS=Candida albicans (strain SC5314 / ATCC MYA-2876) OX=237561 GN=RPS20 PE=1 SV=1	BP:cellular biosynthetic process	Ribosomal_S10	Ribosomal protein S10p/S20e
Q5A8I8	IHD1	Induced during hyphae development protein 1	IHD1_CANAL Induced during hyphae development protein 1 OS=Candida albicans (strain SC5314 / ATCC MYA-2876) OX=237561 GN=IHD1 PE=1 SV=1			

Q5ACU9	NDT80	Transcription factor	NDT80_CANAL Transcription factor NDT80 OS=Candida albicans (strain SC5314 / ATCC MYA-2876) OX=237561 GN=NDT80 PE=1 SV=1	BP:regulation of primary metabolic process	NDT80_PhoG	NDT80 / PhoG like DNA-binding family
Q5AF03	GLX3	Glyoxalase 3	HSP31_CANAL Glyoxalase 3 OS=Candida albicans (strain SC5314 / ATCC MYA-2876) OX=237561 GN=GLX3 PE=1 SV=1	BP:carboxylic acid metabolic process	DJ-1_PfpI	DJ-1/PfpI family
Q5AF44	AHP1	Thioredoxin peroxidase	Q5AF44_CANAL Thioredoxin peroxidase OS=Candida albicans (strain SC5314 / ATCC MYA-2876) OX=237561 GN=AHP1 PE=3 SV=1	BP:response to metal ion	Redoxin;;AhpC-TSA	Redoxin;;AhpC/TSA family
Q96W53	RPS14	Small ribosomal subunit protein uS11	RS14_CANAX Small ribosomal subunit protein uS11 OS=Candida albicans OX=5476 GN=RPS14 PE=3 SV=1	BP:cellular component assembly	Ribosomal_S11	Ribosomal protein S11
Q9P940	TPI1	Triosephosphate isomerase	TPIS_CANAL Triosephosphate	BP:positive regulation of	TIM	Triosephosphate isomerase

			isomerase OS=Candida albicans (strain SC5314 / ATCC MYA-2876) OX=237561 GN=TPI1 PE=1 SV=3	response to stimulus		
Q9UR58	MET15	O-acetylhomoserine O-acetylserine sulphydrylase	Q9UR58_CANAX O-acetylhomoserine O-acetylserine sulphydrylase OS=Candida albicans OX=5476 GN=MET15 PE=2 SV=1	BP:homocysteine biosynthetic process	Cys_Met_Meta_PP;;Aminotran_1_2;;Aminotran_5;;DegT_DnrJ_EryC1	Cys/Met metabolism PLP-dependent enzyme;;Aminotransferase class I and II;;Aminotransferase class-V;;DegT/DnrJ/EryC1/StrS aminotransferase family
Q9URB4	FBA1	Fructose-bisphosphate aldolase	ALF_CANAL Fructose-bisphosphate aldolase OS=Candida albicans (strain SC5314 / ATCC MYA-2876) OX=237561 GN=FBA1 PE=1 SV=2	BP:response to stimulus	F_bP_aldolase	Fructose-bisphosphate aldolase class-II
Q9UVX1	PLB3	Lysophospholipase 3	PLB3_CANAX Lysophospholipase 3 OS=Candida albicans	BP:organophosphate catabolic process	PLA2_B	Lysophospholipase catalytic domain

			OX=5476 GN=PLB3 PE=3 SV=2			
V5UZY2	-	phosphopyruvate hydratase	V5UZY2_CANAX phosphopyruvate hydratase (Fragment) OS=Candida albicans OX=5476 PE=3 SV=1	BP:negative regulation of response to stimulus	Enolase_C;;Enolase_N	Enolase, C-terminal TIM barrel domain;;Enolase, N-terminal domain
V5UZY2	-	phosphopyruvate hydratase	V5UZY2_CANAX phosphopyruvate hydratase (Fragment) OS=Candida albicans OX=5476 PE=3 SV=1	BP:negative regulation of response to stimulus	Enolase_C;;Enolase_N	Enolase, C-terminal TIM barrel domain;;Enolase, N-terminal domain
